# Dual‐Scale Hydration‐Induced Electrical and Mechanical Torsional Energy Harvesting in Heterophilically Designed CNT Yarns

**DOI:** 10.1002/adma.202501111

**Published:** 2025-04-28

**Authors:** Jae Myeong Lee, Wonkyeong Son, Myoungeun Oh, Duri Han, Hyunji Seo, Hyeon Jun Sim, Shi Hyeong Kim, Dong‐Myeong Shin, Chang‐Seok Kim, Seon Jeong Kim, Changsoon Choi

**Affiliations:** ^1^ Department of Electronic Engineering and Biomedical Engineering Hanyang University Seoul 04763 South Korea; ^2^ Department of Biomedical Engineering Konkuk University Chungju 27478 South Korea; ^3^ Textile Innovation R&D Department Korea Institute of Industrial Technology Ansan Gyeonggi‐do 15588 Republic of Korea; ^4^ Department of Advanced Material Engineering Chung‐Ang University Anseong Gyeonggi‐do 17546 Republic of Korea; ^5^ Department of Mechanical Engineering The University of Hong Kong Pokfulam Road Hong Kong 999077 P. R. China; ^6^ Department of Cogno‐Mechatronics Engineering Pusan National University Busan 46241 Republic of Korea

**Keywords:** actuators, energy harvesters, energy harvesting, heterophilic carbon nanotube yarn, water energy

## Abstract

Water holds vast potential for a useful energy source, yet traditional approaches capture only a fraction of it. This study introduces a heterophilically designed carbon nanotube (CNT) yarn with an asymmetric configuration. This yarn is capable of both electrical and mechanical torsional energy harvesting through dual‐scale hydration. Fabricated via half‐electrochemical oxidation, the yarn contains a hydrophilic region enriched with oxygen‐containing functional groups and a hydrophobic pristine CNT region. Molecular‐scale hydration triggers proton release in the hydrophilic region. Consequently, a concentration gradient is established that generates a peak open‐circuit voltage of 106.0 mV and a short‐circuit current of 20.6 mA cm^−2^. Simultaneously, microscale hydration induces water absorption into inter‐bundle microchannels, resulting in considerable yarn volume expansion. This process leads to hydro‐driven actuation with a torsional stroke of 78.8° mm^−1^ and a maximum rotational speed of 1012 RPM. The presented simultaneous harvesting results in electrical and mechanical power densities of 3.5 mW m^−2^ and 34.3 W kg^−1^, respectively, during a hydration cycle. By integrating molecular and microscale hydrations, the proposed heterophilic CNT yarns establish an unprecedented platform for simultaneous electrical and mechanical energy harvesting from water, representing a groundbreaking development for sustainable applications.

## Graphical abstract

Despite the abundance and vast energy potential of water, its efficient utilization is hindered by single‐mode energy conversion. Herein, heterophilic CNT yarns that allow for simultaneous energy harvesting by means of dual‐scale hydration—electrical energy harvesting through proton gradients and mechanical torsional harvesting through microchannel absorption are presented.

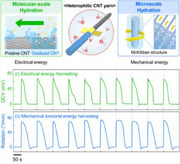

## 1 Introduction

Water is one of the most abundant resources on Earth, and it contains vast amounts of potential energy.^[^ ^1^, ^2^, ^3^, ^4^, ^5^ ^]^ This energy can be converted into electrical and mechanical forms through chemical and physical processes,^[^ ^6^, ^7^ ^]^ which allows us to harness the vast potential of water. Traditional methods such as waterwheels and hydropower have been used to capture energy from water, but they extract a mere fraction of the available energy.^[^ ^8^ ^]^ Recent advancements in carbon‐based nanomaterials, which have high specific surface area, remarkable electrical and mechanical properties, and chemical stability, have opened up new possibilities for more efficient energy capture from water.^[^ ^9^, ^10^, ^11^, ^12^, ^13^ ^]^ Notably, twisted carbon nanotube (CNT) yarn—where millions of individual CNTs are aligned in a single helical direction—has emerged as a promising candidate for water energy harvesting devices, beyond various water‐responsive fibers (e.g., synthetic and bio‐polymers),^[^ ^14^, ^15^, ^16^, ^17^ ^]^ based on the following two key factors: First, at the molecular level, the sp^2^ hybridized carbon atoms in CNTs facilitate active electrical and electrochemical interactions with water molecules, leading to meaningful interface reactions.^[^ ^8^, ^18^, ^19^, ^20^, ^21^ ^]^ Second, at the microscopic level, the one‐chiral McKibben structure and the capillary channels formed between the aligned nano‐bundles promote water absorption and provide sufficient capacity for infiltration.^[^ ^22^, ^23^, ^24^, ^25^, ^26^ ^]^ This multifaceted and unprecedented approach involving chemical and physical interactions holds considerable potential for greatly increasing the overall efficiency of water energy harvesting.

Researchers have developed water‐responsive CNT yarn devices by introducing oxygen‐containing functional groups or incorporating hydrophilic materials. At the molecular level, electrical energy can be harvested through electrochemical interactions between CNT interfaces and charge carriers, such as water molecules or ions.^[^ ^12^, ^18^, ^19^, ^20^ ^]^ For instance, T. Chen's group induced uni‐directional ion movement in an asymmetric CNT composite film to generate electrical energy.^[^ ^27^ ^]^ R. Baughman's group harvested energy by modulating the electric double‐layer capacitance of CNT yarns under mechanical deformation.^[^ ^28^, ^29^, ^30^ ^]^ L. Xu's group developed a CNT‐based water‐induced generator that synergistically integrates electrokinetic and galvanic effects through oxygen‐functionalized CNTs and integrated iron electrodes.^[^ ^31^ ^]^ Later, in 2024, Kim's group further explored the electrochemical behaviors of CNT yarns, demonstrating that structural optimization and interfacial ion dynamics play a crucial role in enhancing their performance for energy harvesting applications.^[^ ^32^, ^33^, ^34^ ^]^

Additionally, infiltration of water into the microchannels within one‐chiral McKibben‐structured CNT yarns can generate mechanical energy.^[^ ^22^, ^23^, ^24^, ^35^ ^]^ S. He's group developed a water‐responsive yarn actuator by using plasma‐treated CNTs,^[^ ^26^ ^]^ while H. Chen's group realized a composite‐based hydro‐actuator by incorporating hydrophilic chitosan into CNTs.^[^ ^36^ ^]^ More recently, in 2023, our group developed electrochemically activated CNT yarn hydro‐actuators with a high torsional actuation factor, demonstrating a large volume expansion due to massive water infiltration.^[^ ^24^ ^]^ Although these pioneering methods have employed mature energy harvesting technologies and demonstrated outstanding performance, their reliance on a singular type of interaction with water limits them to producing only one form of energy. In this light, we expect to achieve a substantial increase in the efficiency of the water energy harvesting systems by inducing diverse interactions between water and CNTs within a single yarn, generating both electrical and mechanical energy.

Herein, we design a heterophilic configuration with asymmetric oxygen content in a single CNT yarn, achieving electrical and mechanical torsional energy harvesting through dual‐scale hydration at the molecular and microscale levels. The heterophilic CNT yarn is prepared by immersing half of the twisted yarn in an electrolyte, followed by selective electrochemical oxidation. This process functionalizes the lower half of the yarn with oxygen‐containing groups, thereby increasing its wettability and responsiveness to water. At the molecular‐scale, hydration triggers the release of protons from the functional groups in the hydrophilic (HPL) region (i.e., oxidized CNTs), which creates a charge carrier concentration gradient toward the hydrophobic (HPB) region (i.e., pristine CNTs). This chemical gradient drives uni‐directional proton diffusion, leading to a hydro‐electric output with a peak open‐circuit voltage (OCV) of 106.0 mV and a peak short‐circuit current (SCC) of up to 20.6 mA cm^−2^. Simultaneously, the HPL region undergoes water absorption‐induced volume expansion in the microchannels formed between the aligned CNT bundles. This microscale hydration induces hydro‐torsional actuation of the yarn, leading to a maximum torsional stroke of 78.8° mm^−1^ and a rotational speed of 1012 RPM. Consequently, we successfully demonstrate simultaneous harvesting of both electrical and mechanical torsional energy from the heterophilic CNT yarn through hydrations at the molecular and microscopic levels.

## 2 Results and Discussion

### 2.1 Simultaneous Energy Harvesting in Heterophilic CNT Yarn through Molecular‐ and Microscale Hydration

**Figure 1:**
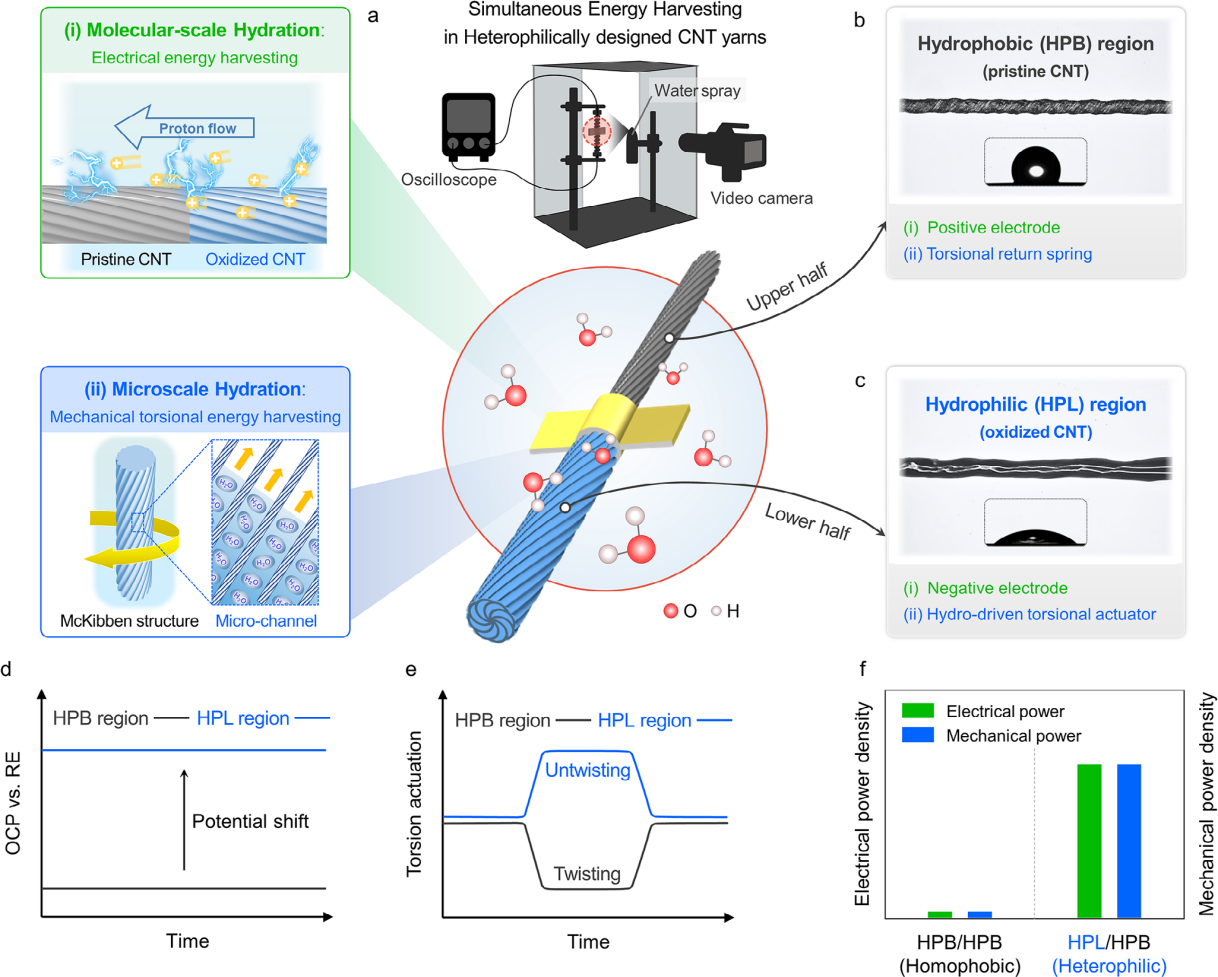
Simultaneous energy harvesting in heterophilically designed carbon nanotube (CNT) yarns through molecular‐ and microscale hydration a) Schematic illustration of the simultaneous energy harvesting in heterophilic CNT yarns, where electrical and mechanical torsional energy occurs at the (i) molecular (upper panel) and (ii) microscopic (lower panel) scales, respectively. A custom‐built apparatus with a water spray setup is used to stimulate hydro‐driven harvesting responses. Electrical output is monitored by an oscilloscope, and mechanical actuation is recorded by a video camera in real‐time. Optical images of the b) hydrophobic (HPB) and c) hydrophilic (HPL) regions depicting their respective roles in simultaneous harvesting. The HPB region (upper half) acts as the positive electrode and provides the torsional return spring, while the HPL region (lower half) serves as the negative electrode and the torsional actuator. Contact angle measurements in both regions confirm the distinct wettability differences. d) Open‐circuit potential shift (versus Ag/AgCl) of the HPB and HPL regions. e) Torsional actuation waveforms of the HPB and HPL regions during a single hydration/dehydration cycle. f) Comparison of the electrical and mechanical power densities between homophobic (HPB/HPB) and heterophilic (HPL/HPB) CNT yarns.

**Figure** **1**a illustrates the experimental setup to investigate simultaneous energy harvesting in heterophilically designed CNT yarns, where both electrical and mechanical torsional energy is generated through dual‐scale hydration processes. In this setup, both ends of the yarn are tethered, and a paddle is attached at the midpoint of the yarn. The electrical output is monitored in real time by using an oscilloscope, and mechanical actuation is recorded using a video camera. Upon hydration by water spray, two distinct processes occur. First, molecular‐scale hydration occurs as the functionalized oxygen species in the HPL region interact with water molecules. This interaction generates a substantial difference in charge carrier concentration (protons) relative to the HPB region, which consists of pristine CNTs with low‐oxygen functional groups.^[^ ^37^, ^38^ ^]^ The resulting imbalance drives uni‐directional proton flows from the HPL to the HPB region, thereby generating an electrical output.^[^ ^39^, ^40^ ^]^ Simultaneously, microscale hydration occurs as water infiltrates the microchannels within the condensed CNT bundles in the HPL region. This water absorption increases the yarn volume, leading to the untwisting of the McKibben structure.^[^ ^23^, ^24^ ^]^ Such a dual‐level interaction across the molecular and microscopic scales facilitates the simultaneous harvesting of electrical and mechanical energy within a single yarn. Figure 1b,c clearly depict the structural and functional differences between the HPB and HPL regions of the heterophilic CNT yarn. The optical image of the HPB region (upper half of the heterophilic CNT yarn) illustrates its hydrophobic nature, as indicated by the higher contact angle (Figure 1b). This region serves as the positive electrode during electrical energy harvesting and as the torsional return spring, ensuring reversible actuation of the yarn after mechanical actuation. By contrast, the electrochemically oxidized HPL region (lower half of the yarn) exhibits increased wettability with a lower contact angle that allows for greater water absorption.^[^ ^24^, ^25^, ^41^ ^]^ This HPL region functions as the negative electrode and drives mechanical actuation upon hydration.

As depicted in Figure 1d, the open‐circuit potentials (OCP) of the HPB and HPL regions were investigated using an Ag/AgCl reference electrode. The HPL region, rich in functional groups, exhibited a notably higher OCP than that of the HPB region. This difference may be attributed to interactions between water molecules and the functional groups, which determine the surface potential.^[^ ^21^ ^]^ The distinct chemical potentials of each region provide the necessary conditions for effective electrical energy harvesting. The electrochemical differences between the HPB and HPL regions are also clearly revealed through cyclic voltammetry (CV) measurements. Oxygen‐containing functional groups provide pseudocapacitance via successive redox mechanisms. Consequently, the CV area of the HPL region was ≈9.2 times greater than that of the HPB region (Figure S1a, Supporting Information). Figure 1e presents the representative torsional actuation waveforms of the HPB and HPL regions during a single hydration/dehydration cycle. Notably, both ends of the yarn are fixed, which ensures that the total number of inserted twists remains constant throughout the cycle. While the HPL region untwists during hydration, the HPB region twists further in response, thereby generating opposing torque. This allows the HPL region to return to its initial state after actuation. In this manner, the HPB region ensures the reversibility and durability of the yarn actuator as a torsional spring. Furthermore, the volumetric responses of the HPB and HPL regions during hydration are clearly distinguished. Unlike the HPB region, the HPL region showed a reversible volume change over 10 repeated hydration/dehydration cycles (Figure S1b, Supporting Information). Figure 1f compares the power densities generated by the homophobic (HPB/HPB) and heterophilic (HPL/HPB) CNT yarns, demonstrating the effectiveness of the asymmetric configuration in simultaneous energy harvesting. This configuration is expected to substantially improve the simultaneous harvesting power density, while a homophobic CNT yarn produces negligible power owing to its inherent hydrophobic nature.

### 2.2 Molecular‐ and Microscale Characterizations of Heterophilic CNT Yarns

**Figure 2:**
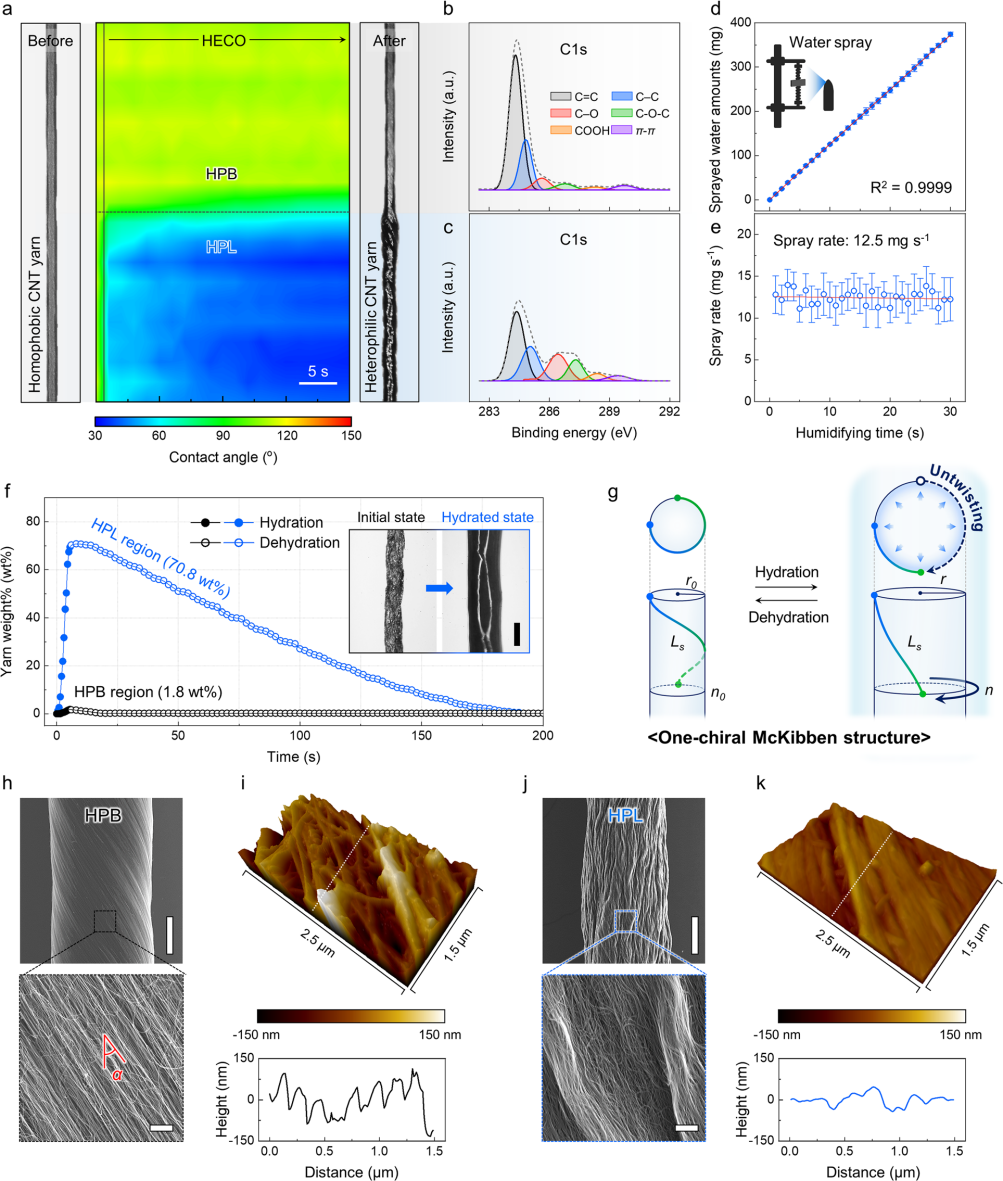
Molecular‐ and microscale characterizations of heterophilic CNT yarns a) Visualization of the surface property in CNT yarn during half‐immersion electrochemical oxidation (HECO) treatment. Optical photographs of a homophobic (left panel) and a heterophilic (right panel) CNT yarn, and a false color mapping representing the contact angle along the CNT yarn length during HECO (middle panel). Deconvoluted C1s X‐ray photoelectron spectroscopy (XPS) spectra of the b) HPB and c) HPL regions. d) Time dependence of the sprayed water amount and e) the spray rate of an ultrasonic humidifier calculated at 1‐second intervals (the number of samples = 4). f) Time dependence of yarn weight% of the HPB and HPL regions during a single hydration/dehydration cycle (inset: optical photographs of the HPL region at initial and fully hydrated states, scale bar = 100 µm). g) Schematic illustration depicting hydration‐induced volume expansion affecting the yarn radius (*r*), individual CNT bundle length (*L_s_*), and the number of twists (*n*) of one‐chiral McKibben structure. h) Scanning electron microscopy (SEM) images of the microscale structure of the HPB region at low (upper panel) (scale bar = 30 µm) and high magnifications (lower panel) (scale bar = 2 µm). i) 3D atomic force microscopy (AFM) mapping (upper panel) and height profile (lower panel) of the HPB region (size = 2.5 × 1.5 µm^2^). Corresponding j) SEM images and k) AFM mapping and height profile of the HPL region.

**Figure** **2** illustrates the differences in chemical compositions and structures of the HPB and HPL regions at the molecular‐ and microscale levels. The fabrication process of the twisted CNT yarn involved three main steps: (i) stacking forest‐drawn CNT sheets, (ii) scrolling them into a cone shape, and (iii) twisting the cone to form the CNT yarn (Figure S2, Supporting Information).^[^ ^42^, ^43^ ^]^ As the twist density increased to 18 turns cm^−1^, the yarn length decreased by ≈40%, while the bias angle (the angle between individual CNTs and the yarn length direction) increased to 35.2° (Figure S3a, Supporting Information). Notably, the yarn diameter decreased by ≈41.7% from 148.3 to 86.3 µm during twisting (Figure S3b, Supporting Information). This morphological transformation suggests that the twisting process helically aligned millions of individual CNTs, thereby compressing inter‐bundle gaps and creating a highly ordered one‐chiral McKibben structure.^[^ ^43^, ^44^, ^45^ ^]^ Subsequently, a half‐immersion electrochemical oxidation (HECO) treatment was applied to fabricate the heterophilic CNT yarn. The lower half of the twisted CNT yarn was immersed in an electrolyte to form an electrochemical cell with a three‐electrode configuration, where the CNT yarn served as the working electrode, Ag/AgCl as the reference electrode, and a Pt mesh as the counter electrode (Figure S4a, Supporting Information). Upon applying a constant voltage of 4 V, the current initially stabilized at a plateau, followed by a sharp decrease (Figure S4b, Supporting Information). This decrease in current density was attributed to the formation of oxygen‐containing functional groups, which disrupted the intrinsic carbon network of the CNTs.^[^ ^24^, ^41^ ^]^ During this process, the lower half of the CNT yarn was oxidized selectively, while the upper half remained untreated. The standard oxidation time was defined as the period up to the observed current drop, and it provided crucial information to determine the endpoint of the HECO process. The current density, oxidation time, and total input energy depended on the applied voltage (Figure S5a, Supporting Information). As the voltage increased from 3 to 6 V, the average current density increased, while the oxidation time decreased inversely. Importantly, the total input energy per treatment remained constant at ≈12.8 kJ g^−1^, irrespective of the voltage applied (Figure S5b, Supporting Information). Unless otherwise stated, the applied voltage was 4 V in all the experiments.

As illustrated in Figure 2a, the surface hydrophilicity changes were observed to evaluate the molecular‐level changes in the CNT yarn during the HECO process. The pristine CNT yarn was initially hydrophobic along its entire length (homophobic CNT yarn), with a contact angle of ≈110.4°. The contact angle of the lower half of the yarn, which was submerged in the electrolyte, decreased gradually during the HECO treatment, reaching 42.2°, indicating increased hydrophilicity. In contrast, the contact angle of the upper half changed negligibly (Figure S6a,b, Supporting Information). As depicted in the right panel of Figure 2a, the HPL region, which was wetted by water, contrasts clearly with the hydrophobic HPB region. To further analyze the chemical composition of the HPB and HPL regions within the heterophilic CNT yarn, X‐ray photoelectron spectroscopy (XPS) was used. The XPS survey spectra revealed prominent peaks corresponding to C1s and O1s at ≈284 and 533 eV, respectively (Figure S7, Supporting Information). The O/C atomic ratio of the HPB region was calculated as 0.03, indicating minimal oxygen content. By contrast, the O/C ratio of the HPL region was significantly elevated at 0.20. These values were obtained using the high‐resolution C1s and O1s spectra of the HPB and HPL regions, respectively. Gaussian peak fitting analysis of the C1s spectrum revealed distinct peaks related to specific chemical bonds, including C═C (284.4 eV), C─C (284.8 eV), C─O (286.4 eV), C─O─C (287.7 eV), COOH (288.8 eV), and π–π* (289.1 eV). In the HPB region, chemical bonds associated with functional groups were scarcely detected, aside from the intrinsic carbon–carbon bonds of CNTs (Figure 2b; Figure S8a, Supporting Information). By contrast, in the HPL region, C─O (hydroxyl) was the most abundant among the oxygen‐containing functional groups, followed by the C─O─C (epoxy) and COOH (carboxylic) groups (Figure 2c). Additionally, the O1s spectrum region exhibited prominent peaks at 532.7 and 531.5 eV, which corresponded to the C─O and COOH bonds, respectively, suggesting that the hydrophilicity increased primarily owing to the formation of C─O bonds (Figure S8b, Supporting Information). These outcomes are consistent with the results of previous studies, thereby demonstrating that the HECO treatment effectively modified the CNTs by introducing hydroxyl functional groups.^[^ ^24^, ^25^, ^41^ ^]^ The mechanical properties of the HPB and HPL regions were evaluated using stress–strain curves (Figure S9, Supporting Information). Compared to the mechanical strength of the HPB region, that of the HPL region decreased by ≈31.3% (from 168.7 to 115.8 MPa) owing to partial disruption of the sp^2^‐hybridized bonds in the CNTs by the oxygen‐containing functional groups introduced during HECO. However, newly formed hydrogen bonds between the functional groups contributed to a slight increase in the maximum strain from 8.0 to 9.1%.^[^ ^46^, ^47^ ^]^

Prior to evaluating yarn hydration, we quantitatively characterized the sprayed water amount. The experiments were conducted in an enclosed chamber (40 × 40 × 40 cm^3^) at 25 °C and 20 RH%. A commercial ultrasonic humidifier, generating droplets of 3–5 µm in size, was employed for spraying. The water amount and spray rate were determined by measuring the weight of the sprayed water at 1‐second intervals. During a 30‐second humidification, the water amount increased linearly, reaching 374.7 mg (Figure 2d). Based on this, the average spray rate was calculated as 12.5 mg s^−1^ (Figure 2e). Unless otherwise noted, the hydration conditions are characterized by the hydration time, with the corresponding sprayed water amount also indicated. The yarn water content was evaluated based on the yarn weight%, calculated as follows:

(1) $$\mathrm{weig}\mathrm{ht}\%\;\;\left(\mathrm{wt}\%\right)=\frac{w_{h}-w_{0}}{w_{h}}\;\times 100$$

where *w_h_* and *w_0_* represent the weight of the hydrated and fully dehydrated yarn.^[^ ^14^ ^]^ As shown in Figure 2f, the HPL region reached a maximum water content of 70.8 wt.% within 5 s of spraying and returned to its initial weight within ≈200 s after spraying was halted. In contrast, the HPB region exhibited negligible changes. When the hydration time increased from 1 to 5 s (corresponding to a sprayed water amount from 12.5 to 62.5 mg), the average water content in the HPL region rose linearly from 2.9 to 68.5 wt.% (Figure S10, Supporting Information). This conspicuous hydration capacity is attributed to the hydrophilicity of the HPL region and the capillary effect within its microchannels. Consistently, the volume change was also exclusive to the HPL region, exhibiting an ≈223% increase, while the HPB region showed negligible variation (Figure S11a, Supporting Information). Such morphological changes are accompanied by a substantial diameter increase and water infiltration, both of which are clearly visible in the inset of Figure 2f and Movie S1 (Supporting Information). Furthermore, the HPL region maintained reversible and consistent volume changes over 50 repeated hydration/dehydration cycles (Figure S11b,c, Supporting Information).

As depicted in Figure 2g, yarn volume expansion induces various morphological and structural changes. Unlike conventional McKibben muscles composed of two oppositely handed helices,^[^ ^48^ ^]^ the CNT yarns are solely composed of single‐handed helices with inextensible CNT bundles (constant bundle length, *L_s_*). This inherent one‐chiral McKibben structure of the CNT yarn originates from the uni‐directional twisting continuously applied during the fabrication process.^[^ ^42^ ^]^ Upon volume expansion (increase in *r*), these helices cause a torque imbalance.^[^ ^42^, ^49^ ^]^ The only way to relieve expansion‐induced internal stress is to generate rotation in the untwisting direction of the yarn (decrease in twisting number, *n*).^[^ ^49^, ^50^ ^]^ Such a distinct behavior resembles that of unbalanced McKibben muscles which provide torsional actuation.^[^ ^51^ ^]^ The detailed mechanism of this torsional actuation is described in the following section.

To clarify the microscale effects of the HECO process on the CNT yarn, morphological differences between the HPB and HPL regions were investigated using scanning electron microscopy (SEM) and atomic force microscopy (AFM). As illustrated in Figure 2h, the HPB region exhibited a distinct bias angle in the low‐magnification surface SEM images and highly aligned nano‐bundles in the magnified view. By contrast, the HPL region exhibited a significantly altered morphology, including densified nano‐bundles, surface buckling, and an indistinct bias angle (Figure 2j). AFM analysis revealed substantial differences in surface height variations between the two regions, further supporting these observations. In the 3D AFM map of the HPB region (Figure 2i), the average surface height difference was 93.8 nm, with pronounced height fluctuations. Meanwhile, the HPL region exhibited a flatter, more uniform surface with a minimal average height variation of 21.1 nm (Figure 2k). All 3D maps were obtained in the noncontact mode over a scanning area of 2.5 × 1.5 µm^2^. The evident morphological changes following HECO treatment caused yarn densification, which led to a decrease in diameter from 86.3 to 71.6 µm and an increase in density from 0.62 to 0.89 g cm^−^^3^ (Figure S12, Supporting Information). Notably, in this process, the internal voids within the CNT yarn were effectively reduced, which resulted in the formation of inter‐bundle microchannels that promoted hydration through the capillary effect.^[^ ^52^, ^53^ ^]^

### 2.3 Electrical and Mechanical Torsional Energy Harvesting Performance of Heterophilic CNT Yarns

**Figure 3:**
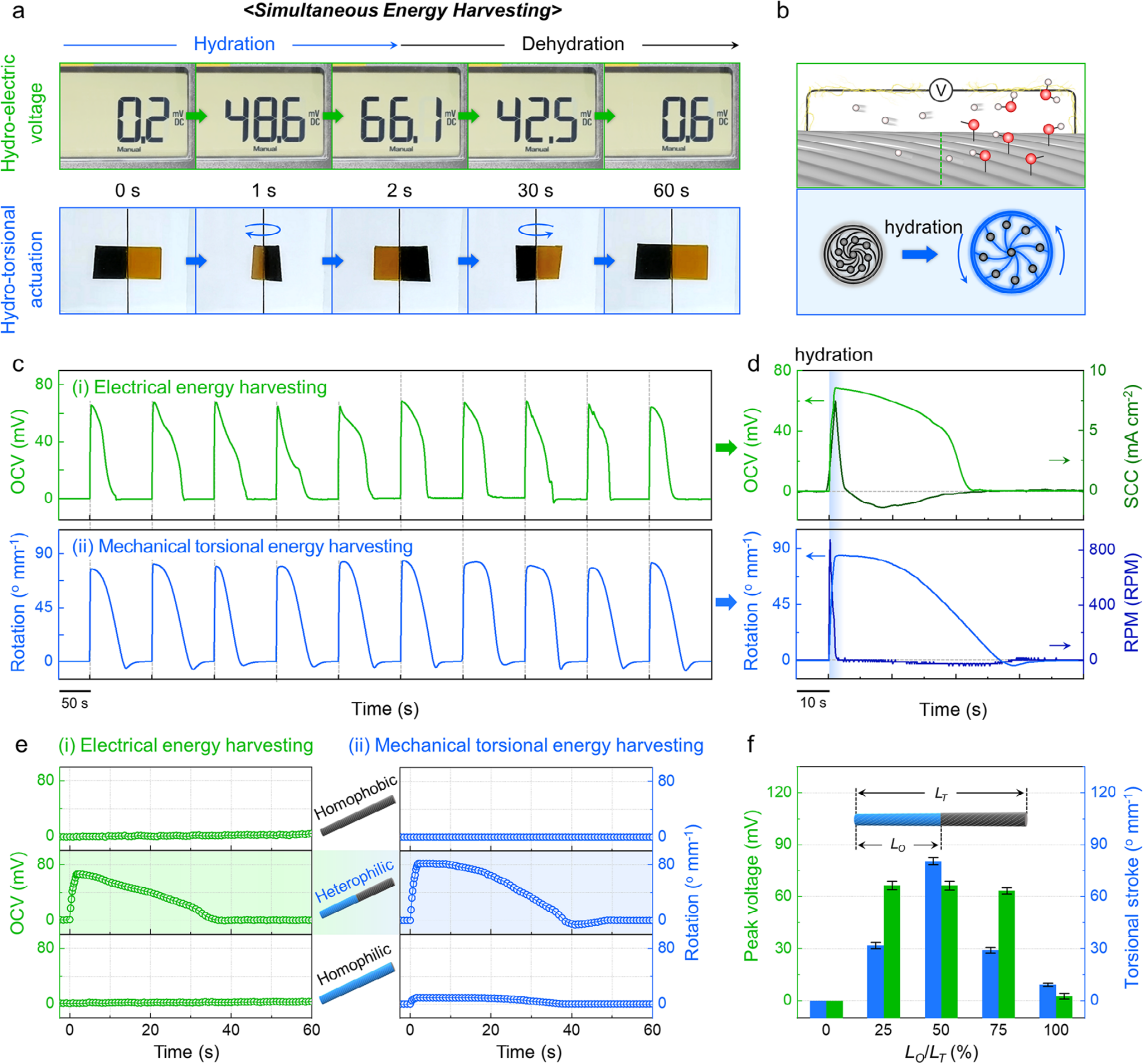
Simultaneous energy harvesting performance of heterophilic CNT yarns a) Sequential photographs demonstrating the simultaneous energy harvesting of hydro‐electric voltage (upper panel) and hydro‐torsional actuation (lower panel) in a single hydration/dehydration cycle. b) Schematic illustrations depicting the mechanisms of (i) electrical and (ii) mechanical torsional energy harvesting. Hydration promotes proton release from functional groups and volume expansion in the HPL region, leading to uni‐directional proton diffusion and the untwisting of the McKibben‐structured yarn, respectively. c) Time dependence of open‐circuit voltage (OCV) (upper panel) and rotation (lower panel) of a heterophilic CNT yarn during 10 consecutive hydration/dehydration cycles. d) Corresponding profiles of OCV and short‐circuit current (SCC) (upper panel), and rotation and revolutions per minute (RPM) (lower panel) during a single hydration/dehydration cycle. e) Comparison of (i) electrical and (ii) mechanical torsional energy harvesting performance of homophobic, heterophilic, and homophilic CNT yarns. The center panel schematically illustrates the corresponding CNT yarn configurations. f) Peak voltage and torsional stroke as a function of the proportion of total yarn length (*L_T_*) to oxidized yarn length (*L_O_*).

In the experimental setup for simultaneous energy harvesting, a heterophilic CNT yarn was placed inside a sealed chamber, where a water spray was directed from a distance of ≈10 cm. Both ends of the yarn were tethered torsionally to prevent unintended untwisting, and a half‐painted paddle weighing 1.9 mg (40 times heavier than the CNT yarn) was affixed at the midpoint to visibly identify rotation. Electrical energy generation was monitored in real‐time by using an oscilloscope, while a video camera was used to record paddle movements. **Figure** **3**a presents sequential images depicting the electrical and mechanical torsional energy harvesting during one hydration/dehydration cycle. Within the first few seconds of hydration, a hydro‐electric voltage is established that peaks at ≈66 mV. Simultaneously, the hydration‐induced yarn volume increase causes the paddle to rotate to an angle of ≈80° mm^−1^. During dehydration, the absorbed water evaporates gradually, and consequently, the potential difference declines steadily. By ≈60 s, the hydro‐electric voltage decreases back to ≈0 mV. Concurrently, the untwisted yarn undergoes reverse rotation, returning to its original position, indicating the full recovery.

Figure 3b illustrated the working mechanism of simultaneous energy harvesting: (i) electrical energy (upper panel) and (ii) mechanical torsional energy (bottom panel). The electrical energy harvesting mechanism of the heterophilic CNT yarn is attributed to its asymmetric structure comprising two regions with different oxygen contents (HPB and HPL regions). Upon molecular‐scale hydration, water molecules are absorbed predominantly in the highly oxidized HPL region, facilitating proton release from oxygen‐containing functional groups, such as ─OH and ─COOH. This proton release establishes a concentration gradient between the HPB and HPL regions, leading to a potential difference within a single CNT yarn. The generated hydro‐electric voltage can be expressed mathematically as follows:

(2) $$\sigma \;\frac{\partial U}{\partial x}=De\frac{\partial n}{\partial x}$$

where *σ* is the electrical conductivity of the absorbed water layer, *∂U/∂x* is the voltage gradient along the yarn length, *D* is the proton diffusion coefficient in the absorbed water layer, *e* is the unit electric charge, and *∂n/∂x* denotes the proton concentration gradient.^[^ ^54^ ^]^ According to Equation (2), this proton‐driven energy harvesting process, powered by the concentration gradient, maintains a potential difference until equilibrium is reached or dehydration occurs. As the proton gradient diminishes, the potential decreases gradually, which is consistent with the results presented in Figure 3b. To further verify the underlying mechanism, a polarity‐switching test was conducted (Figure S13, Supporting Information). Reversing the external measurement connections induced an inversion in the OCV polarity. This directional dependence suggested that protons, released predominantly from the HPL region, diffused uni‐directionally toward the HPB region, driven by the concentration gradient.^[^ ^55^ ^]^ Consequently, the electric potential of the HPL region was consistently higher than that of the HPB region.

The hydration‐induced torsional actuation of CNT yarns can be explained using the single‐helix model. In this model, an individual CNT bundle of length *L_s_*, helically wound around a cylindrical yarn of length *L* and radius *r*, is characterized by a bias angle *α* and twist density *n* (Figure S14, Supporting Information). Given that the geometric parameters *r* and *L* can be expressed as *L_s_* sin *α*/2*πn* and *L_s_* cos *α*, respectively, the volume of the cylindrical yarn *V* can be expressed as follows:^[^ ^44^, ^56^ ^]^

(3) $$V=L\left({L_{s}}^{2}-L^{2}\right)/4\pi n^{2}$$

From this equation, the relationship between twist density and volume change can be derived, as follows:

(4) $${\left(\frac{n}{n_{0}}\right)}^{2}=\frac{V_{0}}{V}\;\frac{L\left({L_{s}}^{2}-L^{2}\right)}{L_{0}\left({L_{s}}^{2}-{L_{0}}^{2}\right)}$$

where *n_0_*, *V_0_*, and *L* *_0_* denote the initial state, while *n*, *V*, and *L* represent the actuated state. This relationship demonstrates that the hydration‐induced volume expansion of the one‐chiral McKibben structured yarn generates torsional actuation.^[^ ^27^ ^]^ Meanwhile, the non‐actuating HPB region (upper half of the yarn) functions as a torsional return spring. During actuation, the untwisting torque in the HPL region is transferred to the HPB region, causing it to twist further. After actuation, the over‐twisted upper half provides an opposing torque, allowing the yarn to return to its initial twisted state, thereby facilitating reversible mechanical actuation.^[^ ^56^, ^57^, ^58^ ^]^

Figure 3c presents the simultaneous energy harvesting performance of the heterophilic CNT yarn over 10 consecutive hydration/dehydration cycles. During each hydration, the OCV value consistently exceeded 60 mV and decreased to 0 mV during dehydration (upper panel of Figure 3b). Simultaneously, the paddle attached to the CNT yarn exhibited repetitive torsional actuation of ≈80° mm^−1^ and subsequently returned to its initial position during the corresponding dehydration (lower panel, Figure 3c). Figure 3d presents detailed profiles of each form of harvested energy within a single cycle. The SCC and rotational speed increased rapidly to peaks of 7.5 mA cm^−2^ and 910 RPM, respectively, as hydration progressed.

To validate the importance of the heterophilicity, the harvesting performance of the homophobic, heterophilic, and homophilic CNT yarns is compared in Figure 3e. Notably, only the heterophilic (HPL/HPB) CNT yarns generated both hydro‐electric voltage and hydro‐torsional actuation simultaneously. For electrical energy harvesting, the homophobic (HPB/HPB) and homophilic (HPL/HPL) structures failed to form a proton concentration gradient owing to the uniform hydration level between both regions.^[^ ^55^ ^]^ In terms of mechanical torsional energy harversting, the homophobic yarn did not interact with water, while in the homophilic structure, hydration‐induced untwisting in the upper region hindered torsional actuation of the lower half of the yarn, and therefore, the mechanical outputs were negligible. According to Figure 3f, the harvesting performance of the heterophilic CNT yarns depends strongly on the proportion of the HPL region to the total yarn length (*L_O_*/*L_T_*). For yarns composed entirely of either the HPB or HPL region (i.e., homophobic and homophilic structures, respectively), no significant electrical or mechanical energy outputs were observed. By contrast, when *L_O_*/*L_T_* was 50%, with the HPL and HPB regions distributed evenly, both hydro‐electric voltage and hydeo‐torsional actuation were maximized. This observation is consistent with Equation (2), which indicates that the generated OCV is governed by the proton concentration gradient and remains independent of the geometric dimensions of the device, such as yarn length or width. Unless otherwise specified, the heterophilic CNT yarns used in this study were prepared with an *L_O_*/*L_T_* ratio of 50%.

### 2.4 Performance Characterization of Dual‐Scale Hydration‐Induced Electrical and Mechanical Torsional Energy Harvesting

**Figure 4:**
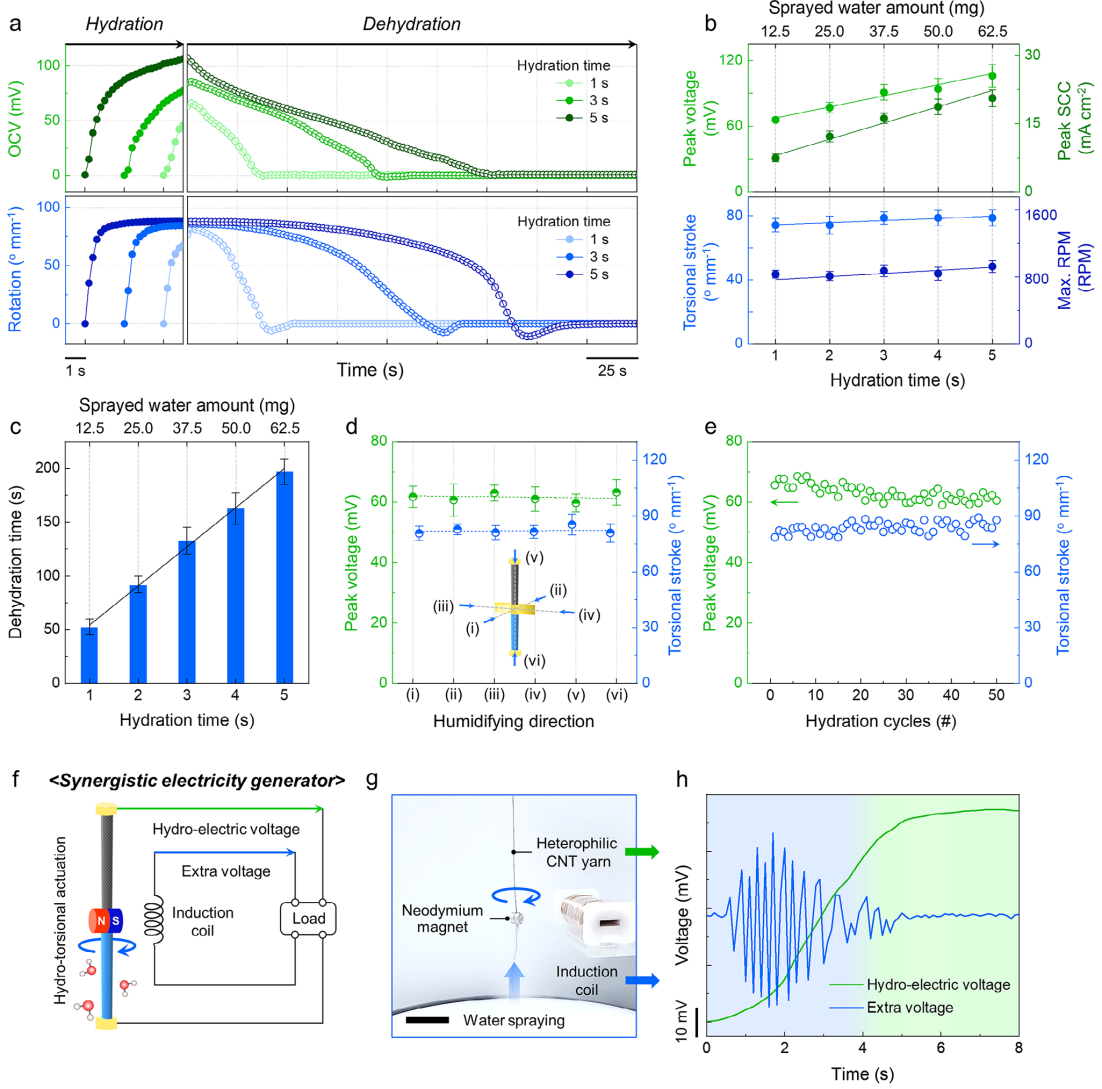
Performance characterization and sustainable application of simultaneous energy harvesting a) OCV (upper panel) and rotation (lower panel) of a heterophilic CNT yarn in hydration and dehydration stages under various hydration times of 1, 3, and 5 s. b) Peak voltage and SCC (upper panel), torsional stroke, and maximum RPM (lower panel) of a heterophilic CNT yarn as a function of hydration time from 1 to 5 s. c) Dehydration time of heterophilic CNT yarns versus hydration time. d) Effect of humidifying direction on the peak voltage and torsional stroke. (inset: schematic illustration depicting the various humidifying directions (i)–(vi) applied to the heterophilic CNT yarn). e) Peak voltage and torsional stroke of heterophilic CNT yarn during 50 repeated hydration cycles. f) Schematic diagram and g) optical image depicting the synergistic electric generator consisting of a heterophilic CNT yarn with a neodymium magnet attached to the bottom end and an induction coil (scale bar = 5 mm). h) Hydro‐electric voltage generated from the heterophilic CNT yarns (green line) and extra voltage in the induction coil (blue line) via electromagnetic induction.

To comprehensively evaluate the total energy harvesting performance of the heterophilic CNT yarn, we systematically characterized its electrical and torsional energy outputs during both the hydration and dehydration stages. The upper and lower panels of **Figure** **4**a depict the OCV and rotation under various hydration times, respectively. With extended hydration, the OCV values exhibited higher peak levels, whereas the rotation exhibited relatively minor rises. Similarly, the SCC and RPM followed comparable trends (Figure S15a,b, Supporting Information). Specifically, as the hydration time increased from 1 to 5 s, the electrical energy harvesting performance—represented by the peak voltage and peak SCC—increased linearly from 65.8 to 106.0 mV and from 7.46 to 20.6 mA cm^−^^2^, respectively (upper panel of Figure 4b). Meanwhile, the mechanical actuation performance, indicated by the torsional stroke and RPM, remained relatively consistent, with a maximum of 78.8° mm^−1^ and 1012 RPM, respectively (lower panel of Figure 4b). These results can be attributed to the following key factors: First, continuous hydration promotes proton release from the oxygen‐containing functional groups in the HPL region, inducing a significant concentration gradient that increases electrical outputs. Meanwhile, the inter‐bundle microchannels within the CNT yarn are rapidly saturated with water during short hydration times, which effectively stabilizes mechanical actuation. Therefore, the hydration time minimally influences the actuation performance. During the subsequent dehydration stage, both the OCV and rotation gradually returned to 0, confirming their reversibility. Notably, the dehydration time was prolonged by ≈3.7 times (from 52.8 to 197.1 s) as the hydration time increased from 1 to 5 s. (Figure 4c). This trend indicates that the hydration and dehydration stages are positively correlated. Such behavior is commonly observed in previous hydro‐driven harvesters and actuators.^[^ ^59^, ^60^, ^61^, ^62^, ^63^ ^]^

Figure 4d depicts the effect of humidifying direction on the energy harvesting performance of the heterophilic CNT yarn. The water spray was applied along six different directions, both parallel and perpendicular to the yarn. Owing to the selective interaction of the HPL region with water molecules, the peak voltage and torsional stroke remained consistent across all humidifying directions. This directional independence suggested that the simultaneous energy harvesting originated from the intrinsic dual‐scale hydration of the heterophilic CNT yarn rather than external environmental conditions. Figure 4e illustrates the cyclic stability for harvesting performance of the heterophilic CNT yarn over 50 hydration and dehydration cycles. Over repeated cycles, the peak voltage and torsional stroke retained 81.6% and 86.7% of their initial values, respectively. This stability was attributed to the consistent charge separation and volume expansion occurring in the HPL region of the heterophilic CNT yarn, even after repeated hydration/dehydration cycles.

Subsequently, the electrical and mechanical power densities generated by the heterophilic CNT yarn through simultaneous hydration were evaluated. The peak voltage was measured across external load resistances ranging from 100 Ω to 10 MΩ to determine the electrical power output (Figure S16a, Supporting Information). The voltage increased progressively with load resistance and the peak electrical power density (*P_e_*) of 3.5 mW m^−2^ was reached at 100 kΩ.

The rotational speed, torque, and mechanical power density, evaluated as a function of the attached paddle's moment of inertia, were also investigated (Figure S16b, Supporting Information). The mechanical power density (*P_m_*) peaked at 34.3 W kg^−1^ when the moment of inertia of the paddle was 1.6 × 10^−11 ^kg m^2^. All measurements were conducted under a consistent hydration time of 5 s, and the electrical and mechanical peak power densities were calculated using the following equations:

(5) $$P_{e}=\frac{V\times I}{unit}=\frac{\;V^{2}}{R}\;\;\times \;\frac{1}{unit}$$

(6) $$P_{m}=\frac{\tau \times \omega }{unit}\;$$

where *V* and *I* represent the measured voltage and current, *R* is the external load resistance, *τ* is the maximum torque, *ω* is the maximum angular velocity, and the *unit* denotes the surface area of the yarn for electrical power density and the yarn weight for the mechanical power density. Meanwhile, we calculated both the peak and average power densities as functions of the sprayed water amount to determine the optimal sprayed water amount. The average power was obtained by dividing the total generated energy by the total cycle time, including both hydration and dehydration periods. Notably, both the electrical and mechanical peak power densities increased with respect to the sprayed water amount (Figure S17a, Supporting Information). This trend was more pronounced in electrical harvesting, as the generated voltage and current increased proportionally with the applied water. On the other hand, the average power per cycle, which accounted for the dehydration time, exhibited an opposite trend (Figure S17b, Supporting Information). This decrease was particularly distinct in mechanical energy harvesting, as the mechanical performance was relatively stable while the dehydration time was significantly prolonged upon spraying (Figure 4b,c). Considering these results, the optimal spraying amount varies depending on the desired output. A larger amount of sprayed water is preferable for maximizing peak power density, whereas a smaller amount is effective for enhancing average power density.

In order to demonstrate the potential application of simultaneous energy harvesting, we present two application scenarios (Figure S18, Supporting Information). These application scenarios are designed to employ the individual energy generated from a heterophilic CNT yarn, with each focusing on a distinct device: an electrical energy harvester (Scenario I) and a mechanical actuator (Scenario II). Scenario I (left panel) illustrates the potential for a sustainable energy harvester. This system enables synergistic electricity generation by inducing an extra voltage through torsional actuation, along with the hydro‐electric voltage from the CNT yarn. Scenario II (right panel) suggests the potential use of the system as a power‐enhanced hydro‐actuator. This design employs hydro‐electric voltage for electric heating to accelerate recovery, leading to a significant enhancement in actuator power density.

Figure 4f exhibits a schematic diagram of a synergistic electric generator that integrates two distinct forms of energy into a single electrical output. The system consists of a 4 cm‐long heterophilic CNT yarn with a neodymium magnet attached at its lower end, and an induction coil (Figure 4g). Upon hydration, the hydro‐electric voltage generated from the CNT yarn gradually increases over ≈5 s and stabilizes at a peak value of 37.3 mV. Simultaneously, a voltage was induced in the induction coil by the rotation of the magnet, reaching a peak amplitude of 13.6 mV within 5 s (Figure 4h). The proposed system effectively compensates for a temporal power deficit that occurs during the initial hydration stage due to the voltage stabilization period. This is achieved by supplying an extra voltage via electromagnetic induction. Such a synergistic generation strategy not only enhances the efficiency of water energy harvesting but also presents a new possibility for sustainable electricity generation. To the best of our knowledge, such an approach has rarely been reported in previous literature.

The second scenario presents a power‐enhanced hydro‐actuator that utilizes hydro‐electric voltage generated by a heterophilic CNT yarn as an electric heating source. Naturally, dried hydro‐actuators typically suffer from delayed recovery after actuation and degraded average power density, caused by slow dehydration resulting from their hydrophilicity.^[^ ^25^ ^]^ In contrast, electric heating by hydro‐electric voltage can promote water evaporation and significantly accelerate recovery time (Figure S19a, Supporting Information). Accordingly, this approach is expected to substantially enhance the power density of hydro‐actuators (Figure S19b, Supporting Information). However, this system would require additional power management units, including rectification, storage, and control, which are beyond the scope of this study. Therefore, continued research is essential for the comprehensive development and practical implementation of such systems.

## 3 Conclusion

In summary, we developed a heterophilic CNT yarn with an asymmetric structure capable of electrical and mechanical torsional energy harvesting through dual‐scale hydration. By means of HECO treatment, oxygen‐containing functional groups were introduced selectively into the lower region, while the hydrophobicity of the upper region was effectively preserved. During molecular‐scale hydration, the protons released from the functional groups in the HPL region established a concentration gradient between the HPL and HPB regions. Owing to this gradient, a peak voltage of 106.0 mV and a peak SCC of 20.6 mA cm^−2^ were generated, demonstrating electrical energy harvesting from water. Simultaneously, during microscale hydration, water absorption into the inter‐bundle microchannels in the HPL region induced significant yarn volume expansion. Driven by this volume change, a torsional stroke of 78.8° mm^−1^ and a maximum rotational speed of 1012 rpm were generated, representing hydro‐driven mechanical torsional energy harvesting. This simultaneous harvesting led to electrical and mechanical power densities of 3.5 mW m^−2^ and 34.3 W kg^−1^, respectively. Although the harvesting performance could be relatively lower than that of previously reported yarn‐ or fiber‐based hydro‐electric harvesters and hydro‐actuators (Table S2, Supporting Information), a notable achievement of this work lies in the simultaneous harvesting of both electrical and mechanical energy from a single hydro‐driven source. Furthermore, we also demonstrated that the two distinct forms of energy can ultimately be integrated into a unified electrical output. The proposed simultaneous energy harvesting from heterophilic CNT yarn presents a new possibility for efficient water energy conversion and provides a meaningful foundation for the development of next‐generation energy harvesting devices for sustainable applications.

## 4 Experimental Section

### Preparation of Twisted CNT Yarn

To fabricate the twisted CNT yarn, a multi‐walled carbon nanotube (MWNT) forest with a height of 320 µm (A‐Tech System Co., Korea) was used. Five CNT sheets, each measuring 2 cm in width and 10 cm in length, were drawn from the forest and stacked sequentially. Both ends of the stacked sheet were attached to adhesive carbon tape, and the stack was carefully rolled into a compact cylinder with a diameter of 5 mm for cone spinning. The prepared CNT cylinder was suspended vertically from the motor tip of a custom‐built twisting machine (K6G3C, GGM), and a 5‐g load was applied to maintain tension. Subsequently, a twist of 18 turns cm^−1^ was introduced gradually, resulting in the fabrication of the twisted CNT yarn. To realize stable electrical connections, a copper wire (diameter: 180 µm) was connected to the twisted CNT yarn using silver paste, and both ends were insulated with epoxy adhesive. This setup was used for the HECO treatment and subsequent electrical signal measurements.

### Electrochemical Oxidation Treatment

To realize a heterophilic structured CNT yarn, HECO treatment was applied using a three‐electrode system consisting of the CNT yarn as the working electrode (W.E.), an Ag/AgCl reference electrode (R.E.), and a platinum mesh counter electrode (C.E.). Only the lower half of the twisted CNT yarn was submerged in an electrolyte solution (0.1 m Na₂SO₄). Then, a constant voltage of 4 V was applied to the CNT yarn using an electrochemical analyzer. This process selectively assigned localized hydrophilic properties to the lower half of the yarn. The treatment time was controlled until the point where a drastic decrease in current density was observed during the HECO process.

### Characterization

Optical images were captured using a digital camera (D750, Nikon, Japan), and magnified surface images were recorded using a scanning electron microscope (S‐4600, Hitachi, Japan) operated at an accelerating voltage of 15–20 kV and a working distance of ≈40 mm. The detailed yarn surface morphology was characterized using an AFM (XE‐100, Park Systems, Korea) in the non‐contact mode with a cantilever length of 125 µm, width of 30 µm, force constant of 42 N m^−1^, and resonant frequency of 204—497 kHz. Surface mapping was performed over an area of 1 × 2 µm^2^ with a scan frequency of 0.4 Hz at 256 pixels. The chemical composition of the yarn was determined using XPS (ESCALAB 250XI, Thermo Scientific, USA). Resistance measurements were performed using a multimeter probe (17B+, FLUKE, USA). Thermal images were captured using a near‐infrared camera (XI400, Optris) to assess heat distribution. All electrochemical performance evaluations were conducted using an electrochemical analyzer (Vertex EIS, Ivium). The mechanical properties of the yarn were measured using a universal testing machine (Instron 5966, Instron, Norwood, USA) at a strain rate of 0.5 mm min^−1^. The voltage and current generated during electrical energy harvesting were monitored using an oscilloscope (Tektronix, DPO4014B, USA), and the mechanical movements were recorded using a video camera (RX10 IV, Sony, Japan).

### Measurement of Simultaneous Energy Harvesting

The experimental setup for simultaneous energy harvesting was designed to monitor both electrical and mechanical torsional energy harvesting in real time. The CNT yarn was tethered securely at both ends to prevent unintended rotations and suspended vertically inside a custom‐built enclosed chamber (40 × 40 × 40 cm^3^), where temperature and humidity were controlled to 25 °C and 20 RH%. During hydration, water was sprayed on the yarn from a distance of ≈10 cm using a fine mist nozzle. For electrical energy harvesting measurements, the yarn was connected to an oscilloscope (Tektronix, DPO4014B, USA). OCV and SCC were monitored in real time throughout the hydration and dehydration cycles. For mechanical actuation measurements, a half‐painted paddle weighing 1.9 mg was attached to the center of the yarn. This paddle served as an optical marker to conveniently identify torsional actuation. Paddle movement was recorded using a video camera. Rotational movements were determined quantitatively by analyzing the changes in the projected paddle width through frame‐by‐frame analysis of the recorded video. This allowed for precise measurement of the rotation angle over time. Each experiment was repeated five times, and the average and standard deviation values were reported to account for variability in the measurements.

## Conflict of Interest

The authors declare no conflict of interest.

## Supporting information



Supporting Information

Supplemental Movie 1

## Data Availability

The data that support the findings of this study are available from the corresponding author upon reasonable request.
