# Supplementary material for: Dual‐Scale Hydration‐Induced Electrical and Mechanical Torsional Energy Harvesting in Heterophilically Designed CNT Yarns
Source: Adv Mater. 2025 Apr 28;37(28):2501111. doi: 10.1002/adma.202501111 (PMC12272001; doi:10.1002/adma.202501111)
Supplement: Supplementary file 1 — Supporting Information [file ADMA-37-2501111-s002.docx]

Supporting Information

**Dual-Scale Hydration-Induced Electrical and Mechanical Torsional Energy Harvesting in Heterophilically Designed CNT Yarns**

Jae Myeong Lee^§^, Wonkyeong Son^§^, Myoungeun Oh, Duri Han, Hyunji Seo, Hyeon Jun Sim, Shi Hyeong Kim, Dong-Myeong Shin, Chang-Seok Kim, Seon Jeong Kim*, and Changsoon Choi*

**
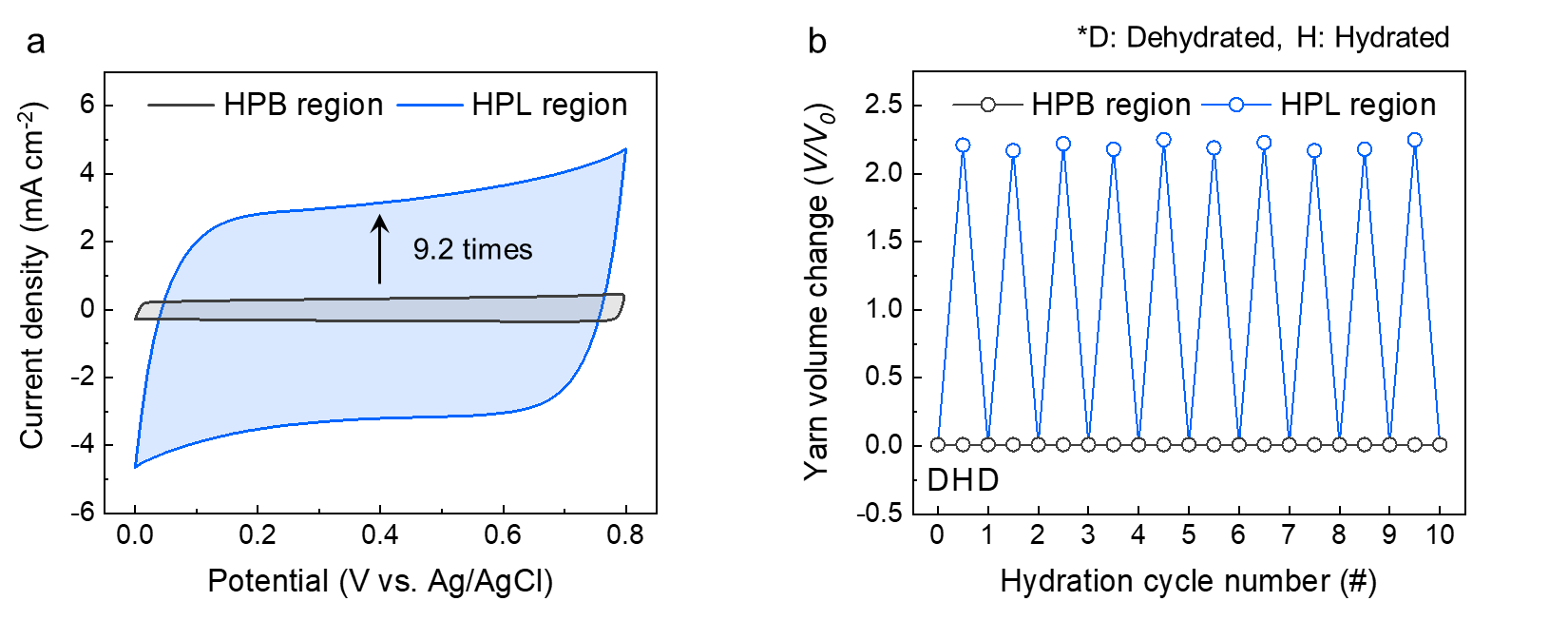
**

**Figure S1** Comparison of a) cyclic voltammetry (CV) curves of the hydrophobic (HPB) and hydrophilic (HPL) regions at a scan rate of 100 mV s^-1^. b) Yarn volume changes of the HPB and HPL regions over 10 repeated hydration/dehydration cycles. *V_0_* and *V* denote the yarn volumes at initial (fully dehydrated) and hydrated state, respectively.


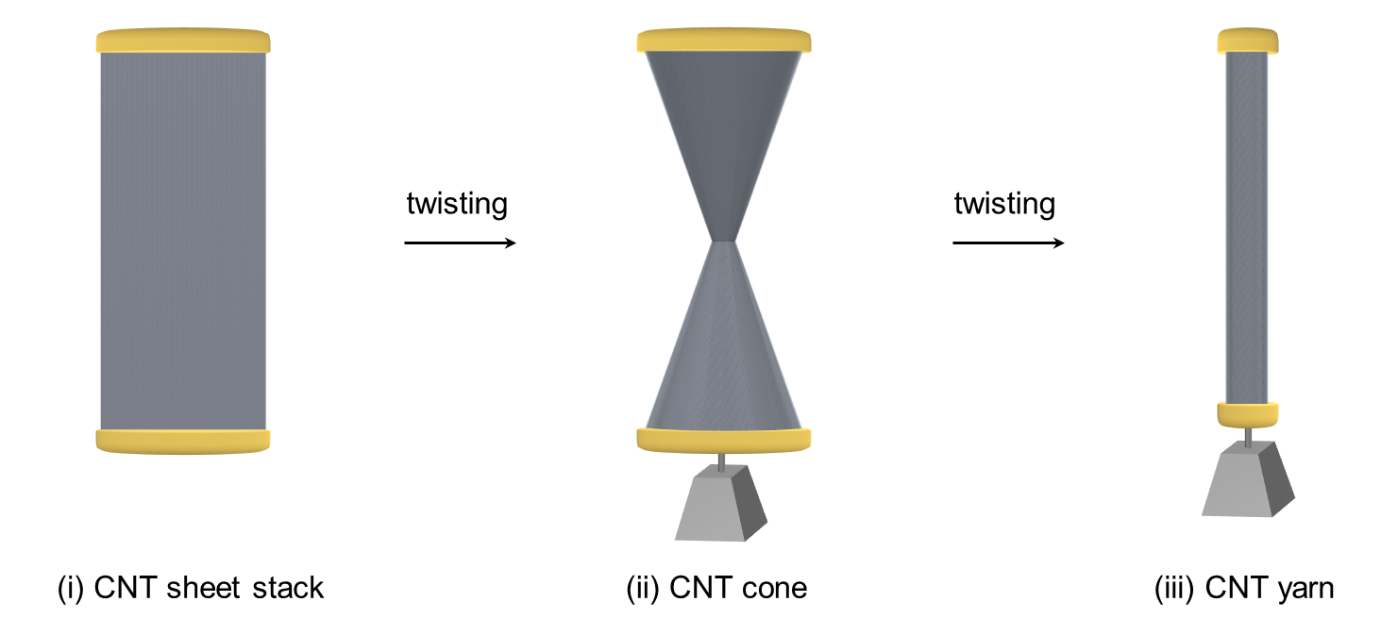


**Figure S2** Schematic illustrations depicting the fabrication processes of twisted CNT yarn; (i) stacking of forest-drawn CNT sheets, (ii) scrolling of stacked sheets into a cone shape, and (iii) twisting of the cone to form CNT yarn.


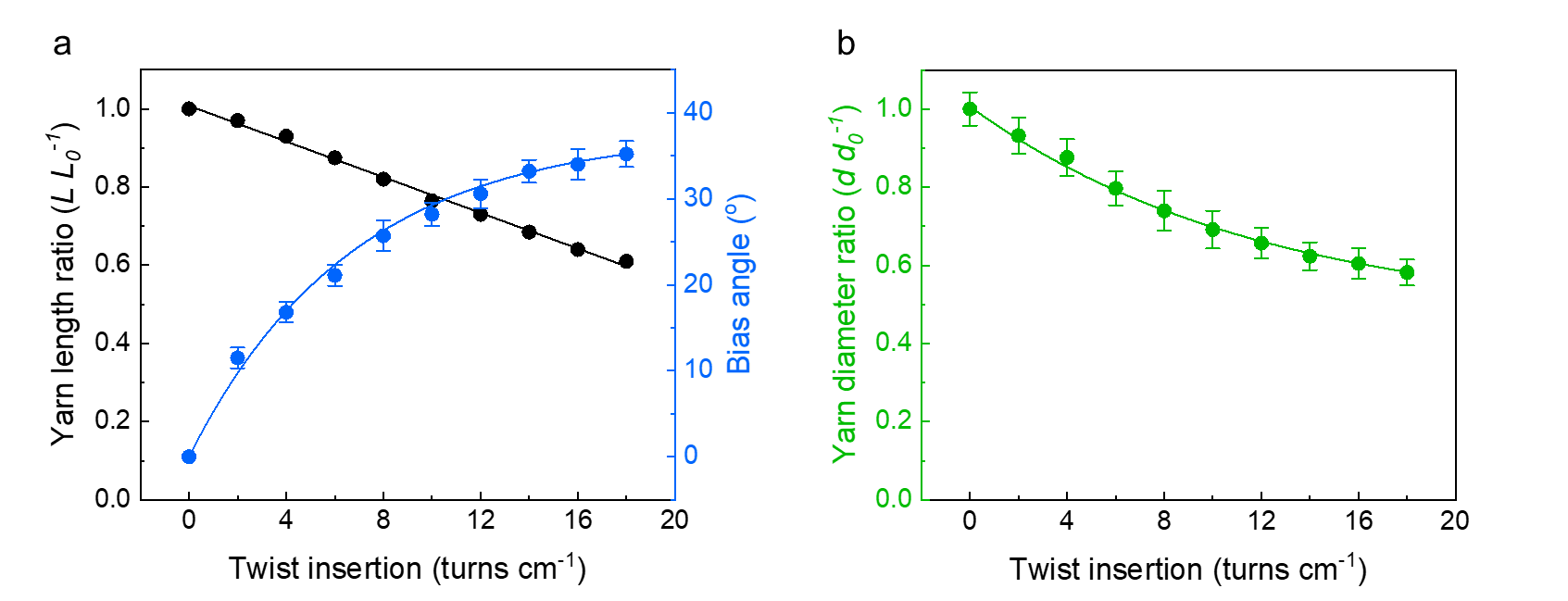


**Figure S3** a) Yarn length ratio (*L L_0_^-1^*) and bias angle, and b) yarn diameter ratio (*d d_0_^-1^*) of twisted CNT yarn during twist insertions, where *L_0_* and *L* denote the initial and twisted CNT yarn length and *d_0_* and *d* denote the initial and twisted CNT yarn diameter, respectively (the number of samples = 5).

**
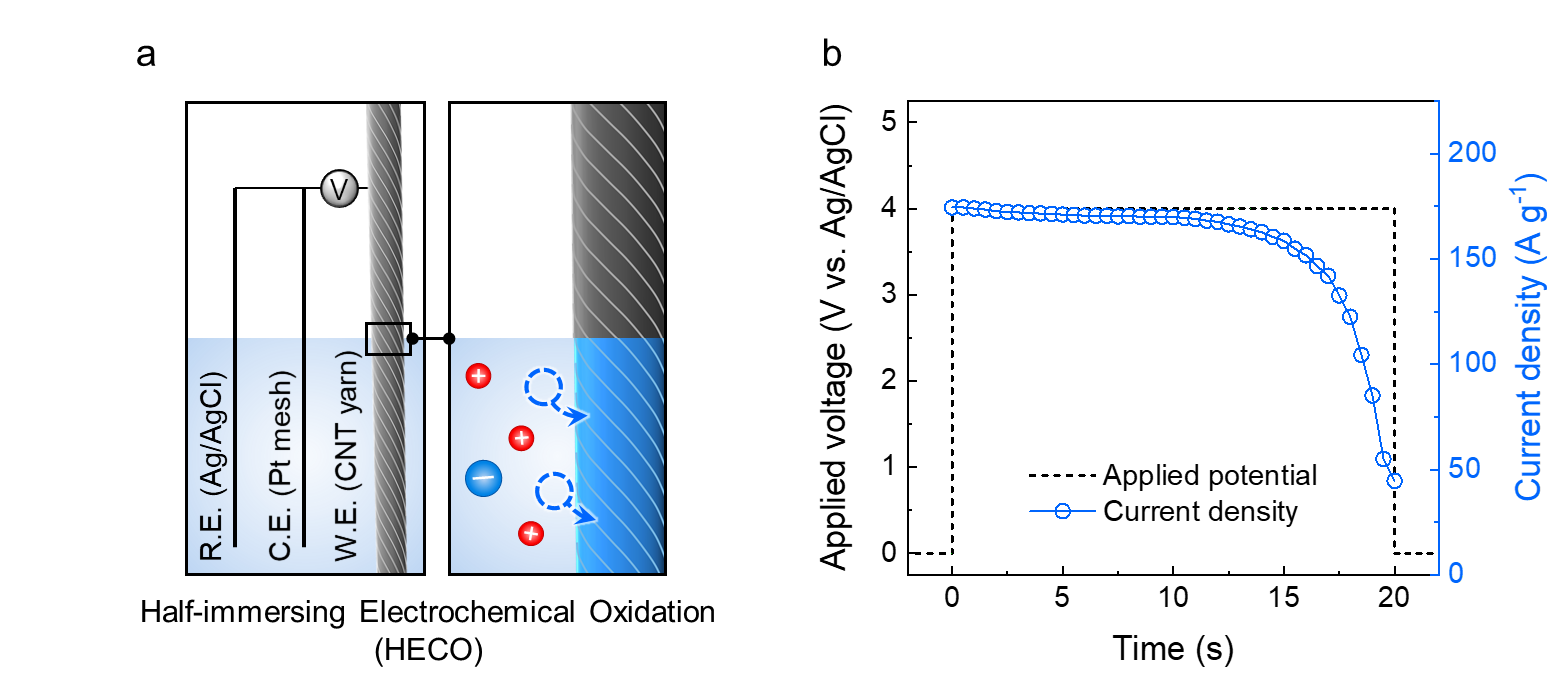
**

**Figure S4** a) Schematic illustration depicting the experimental setup for half-immersing electrochemical oxidation (HECO) treatment using a three-electrode system consisting of the CNT yarn as the working electrode, an Ag/AgCl as the reference electrode, a platinum mesh as the counter electrode, and 0.1 M Na₂SO₄ solution as the electrolyte. Only the half of the CNT yarn is immersed into the electrolyte. b) Time dependence of the applied voltage (vs. Ag/AgCl) and current density (normalized to the yarn mass immersed into the electrolyte).


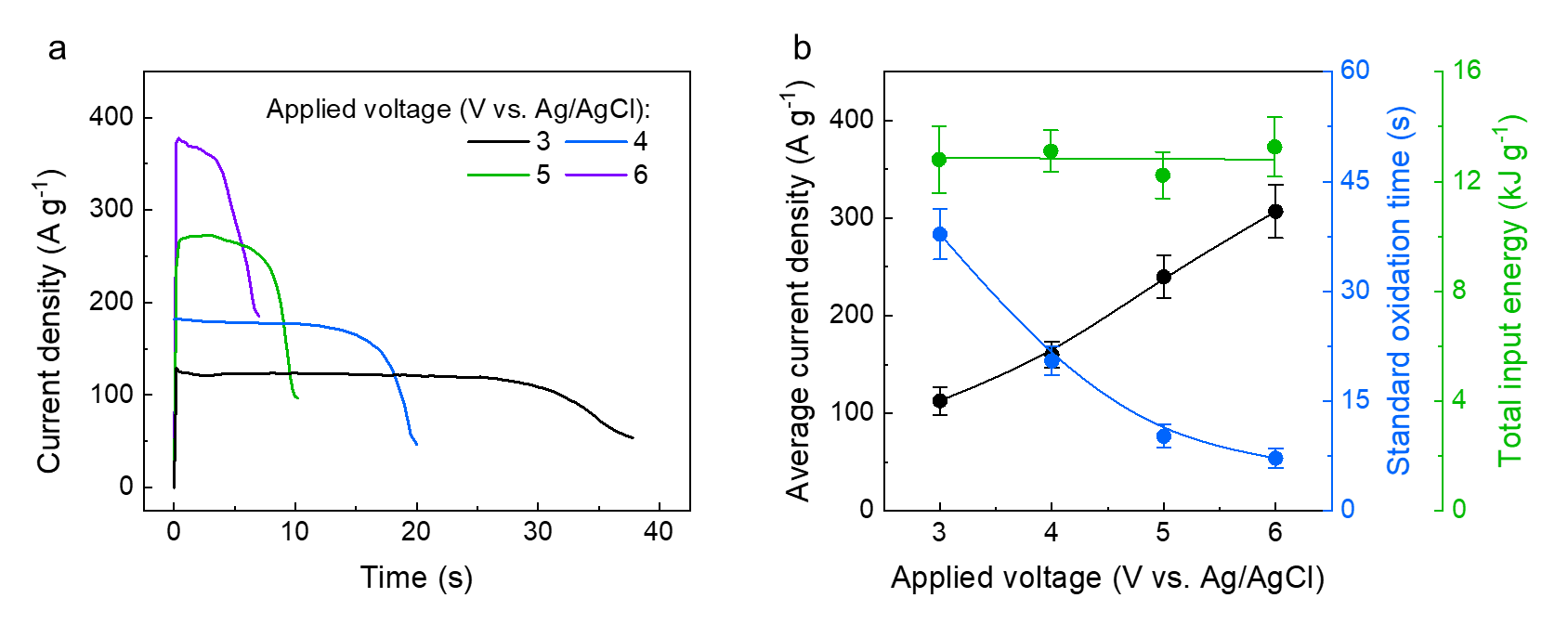


**Figure S5** a) Time dependence of current density under various applied voltages of 3–6 V (vs. Ag/AgCl). b) Average current density, standard oxidation time, and total input energy as a function of applied voltage (the number of samples = 5).

**
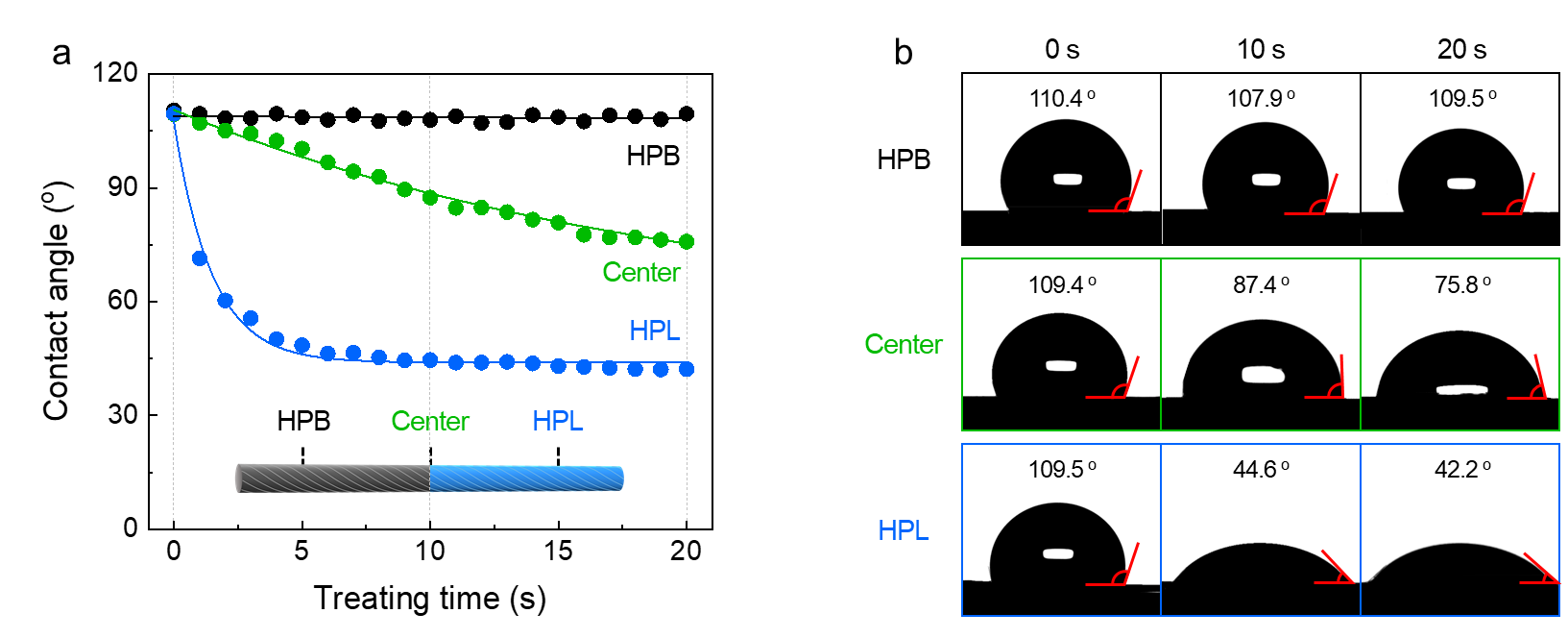
**

**Figure S6** a) Contact angle changes and b) corresponding contact angle images at the HPB, center, and HPL regions within the heterophilic CNT yarn during HECO treatment.


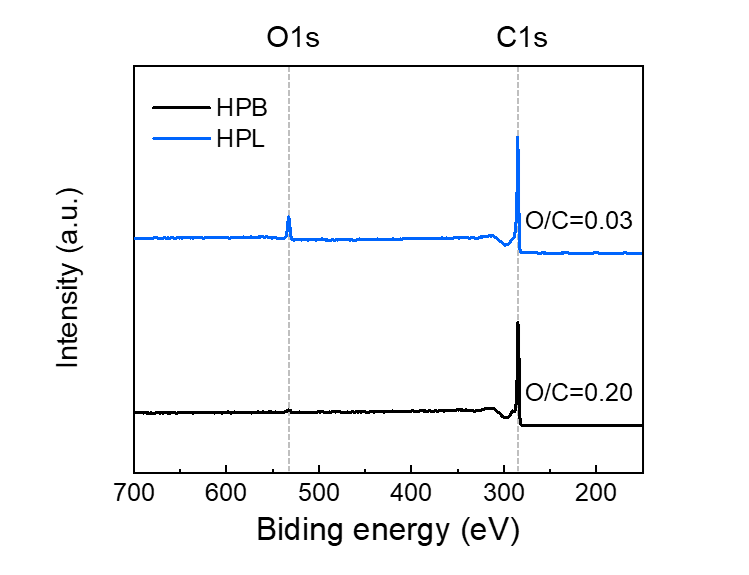


**Figure S7** X-ray photoelectron spectroscopy (XPS) spectrum of the HPB and HPL regions within the heterophilic CNT yarn.

**
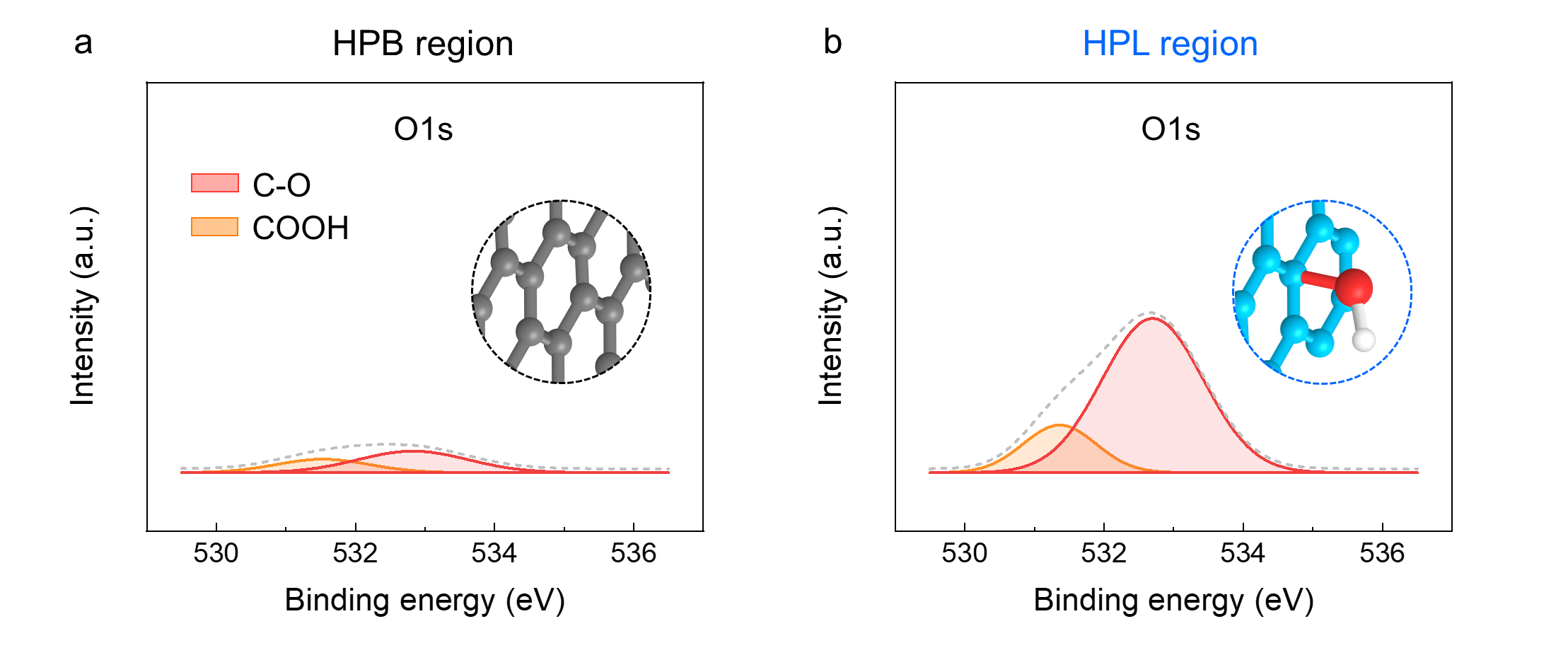
**

**Figure S8** Deconvoluted O1s XPS spectra of the a) HPB and b) HPL regions within the heterophilic CNT yarn.

**
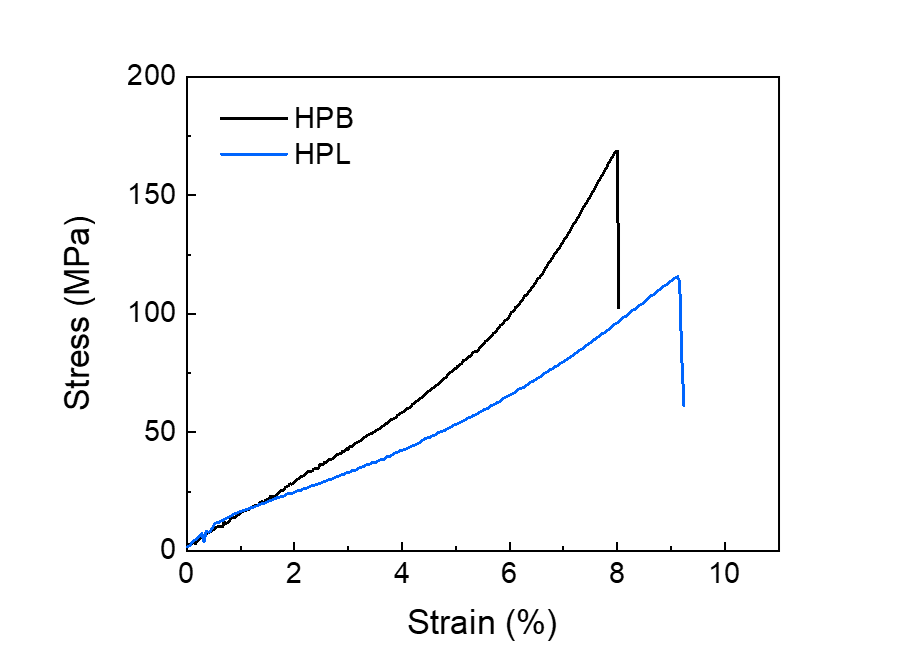
**

**Figure S9** Stress-strain curves of the HPB and HPL regions in the heterophilic CNT yarn.


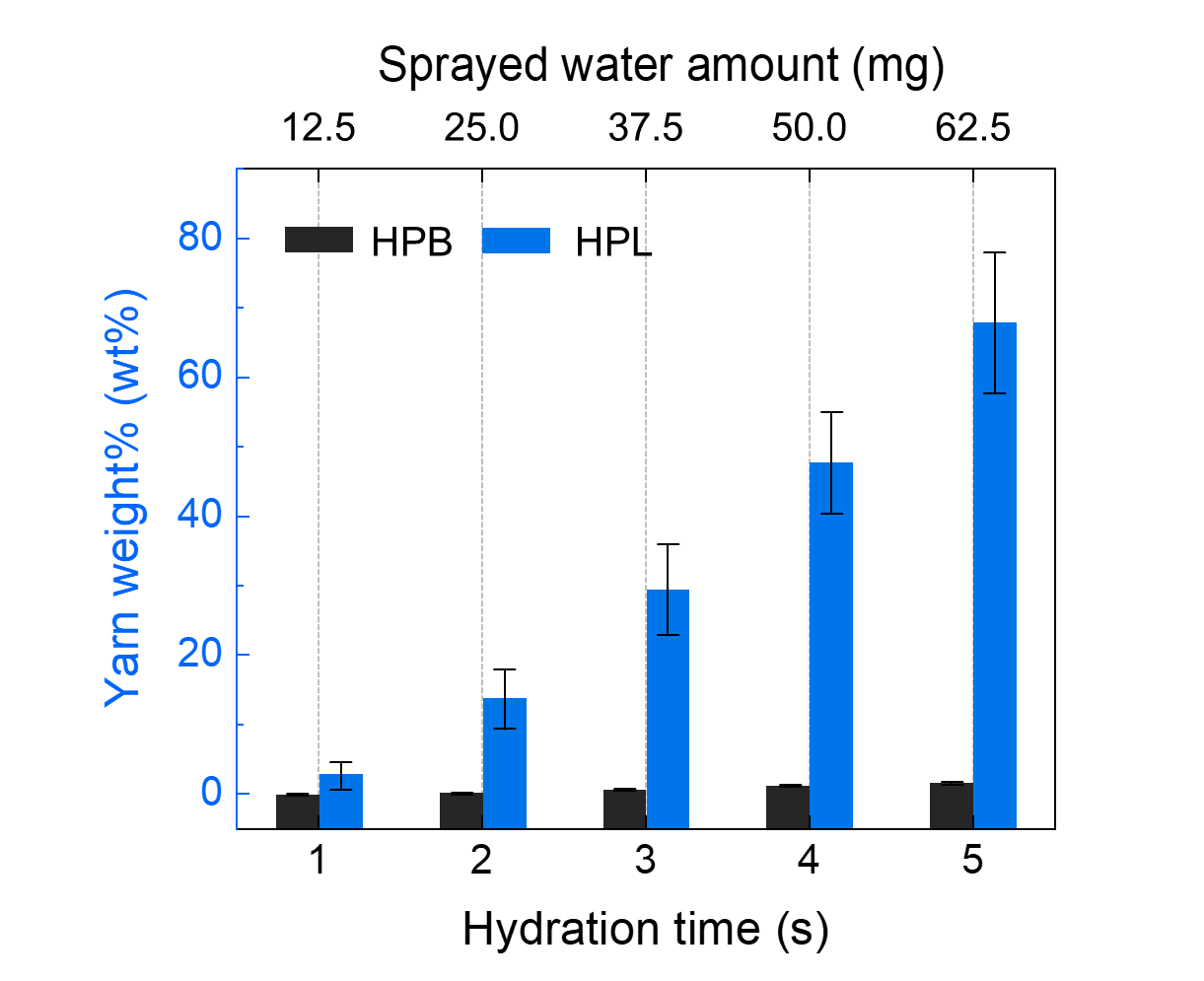


**Figure S10** Yarn weight% of HPB and HPL regions as a function of a hydration time from 1 to 5 s (corresponding to the sprayed water amount from 12.5 to 62.5 mg) (the number of samples = 5).


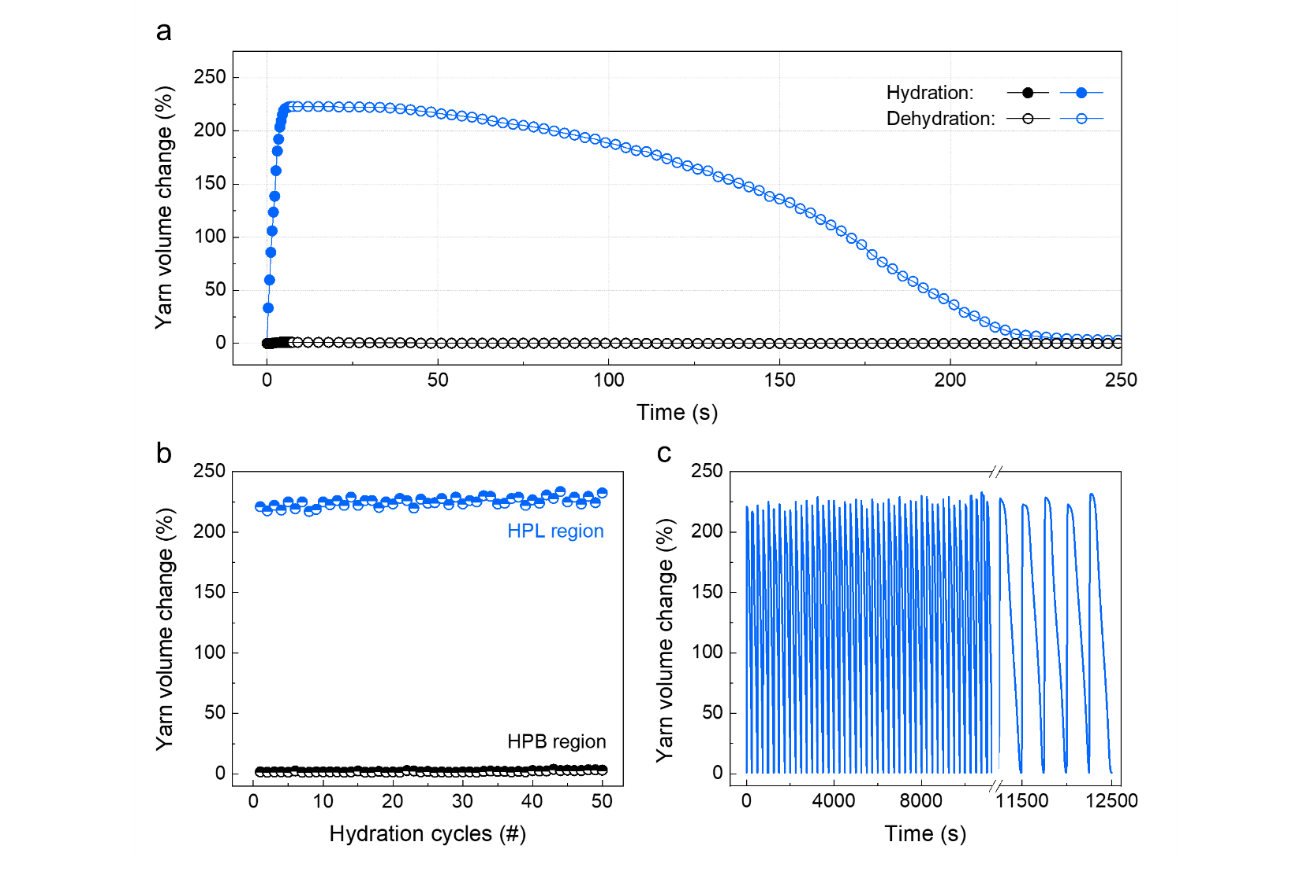


**Figure S11** a) Time dependence of volume changes of the HPB and HPL regions during a single hydration/dehydration cycle. b) Volume changes of the HPB and HPL regions and c) time dependence of volume changes of the HPL region over 50 repeated hydration/dehydration cycles.

**
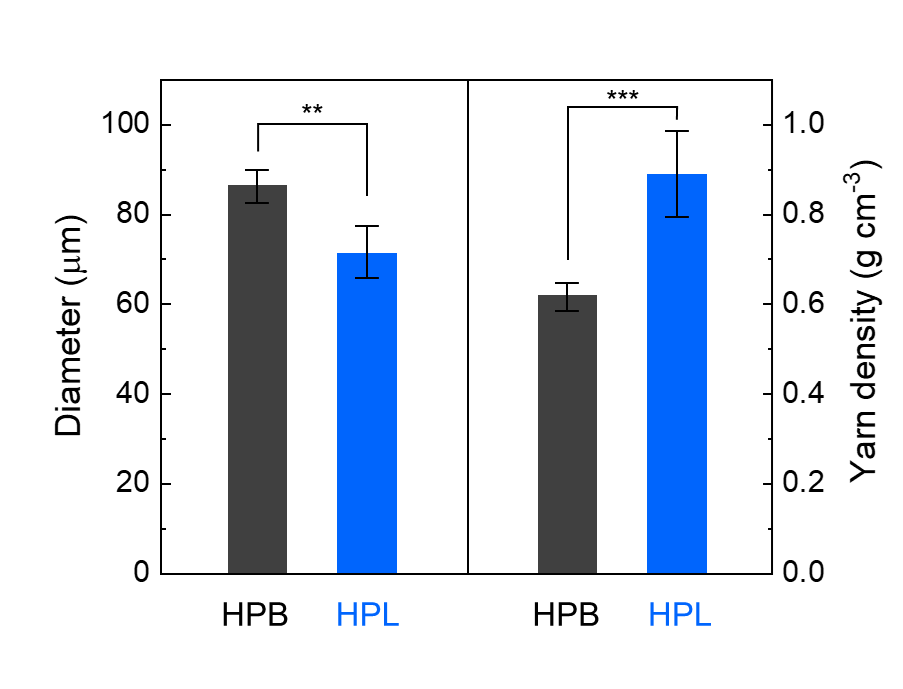
**

**Figure S12** Diameter and density of the HPB and HPL regions in the heterophilic CNT yarn (number of samples = 5).

**
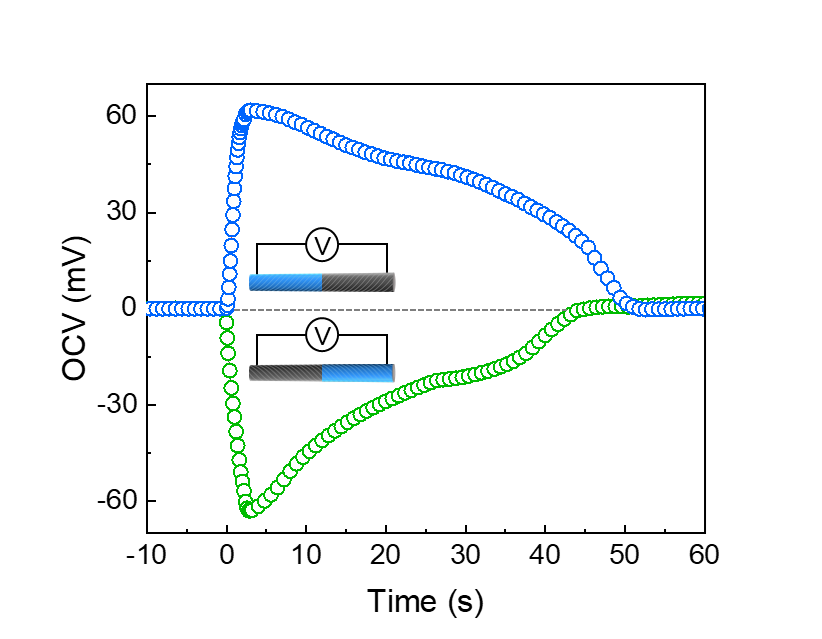
**

**Figure S13** Polarity-switching behavior of the hydro-electric voltage generated by the heterophilic CNT yarn during a single hydration/dehydration cycle. The sign of the open-circuit voltage (OCV) reversibly switched depending on the electrode connection direction.


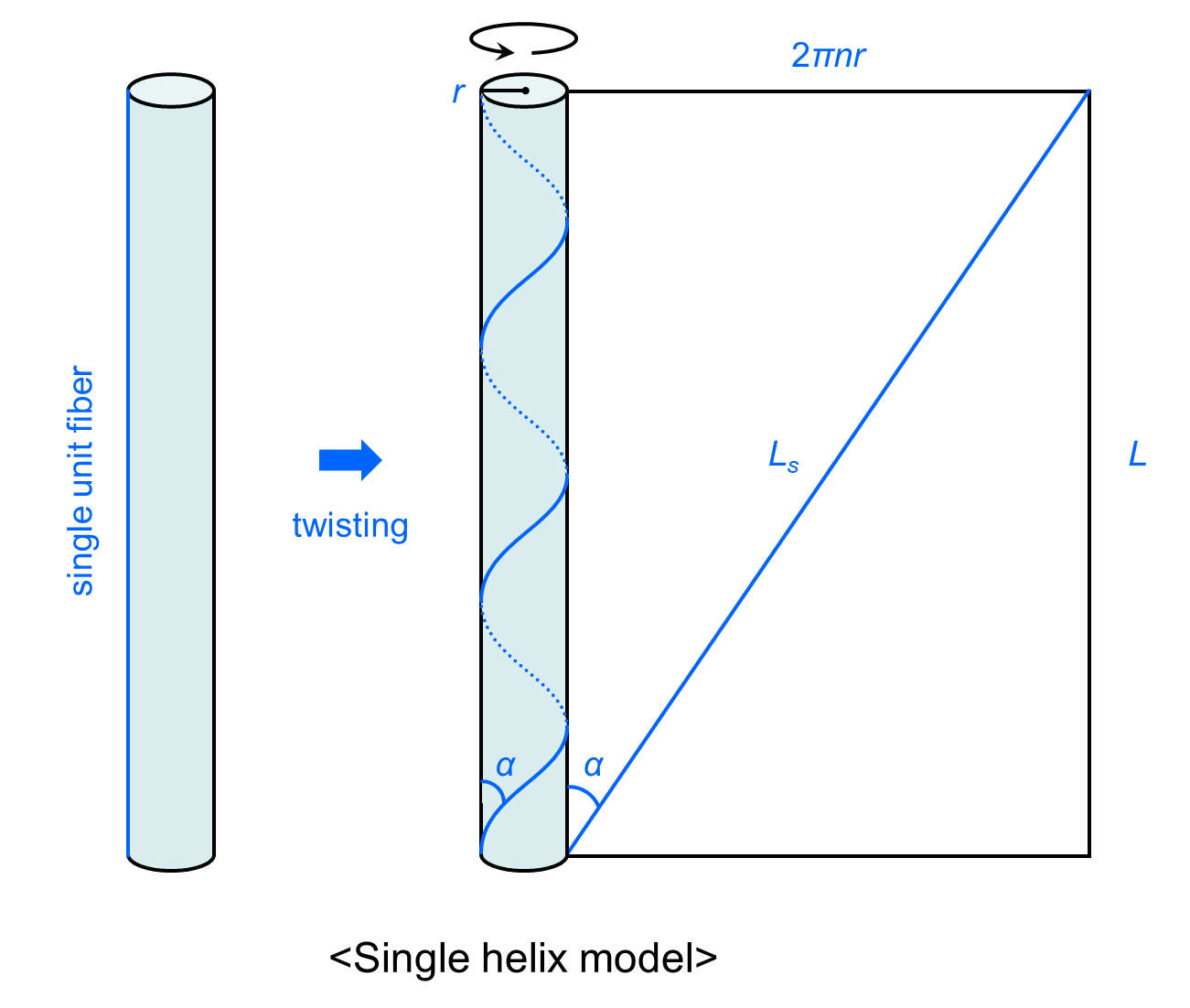


**Figure S14** Schematic illustration depicting the single helix model of twisted CNT yarns. The blue line represents a length of individual CNT bundle (*L_s_*). The yarn radius (*r*), yarn length (*L*), bias angle (*α*), and number of twists (*n*) depend on yarn volume change.


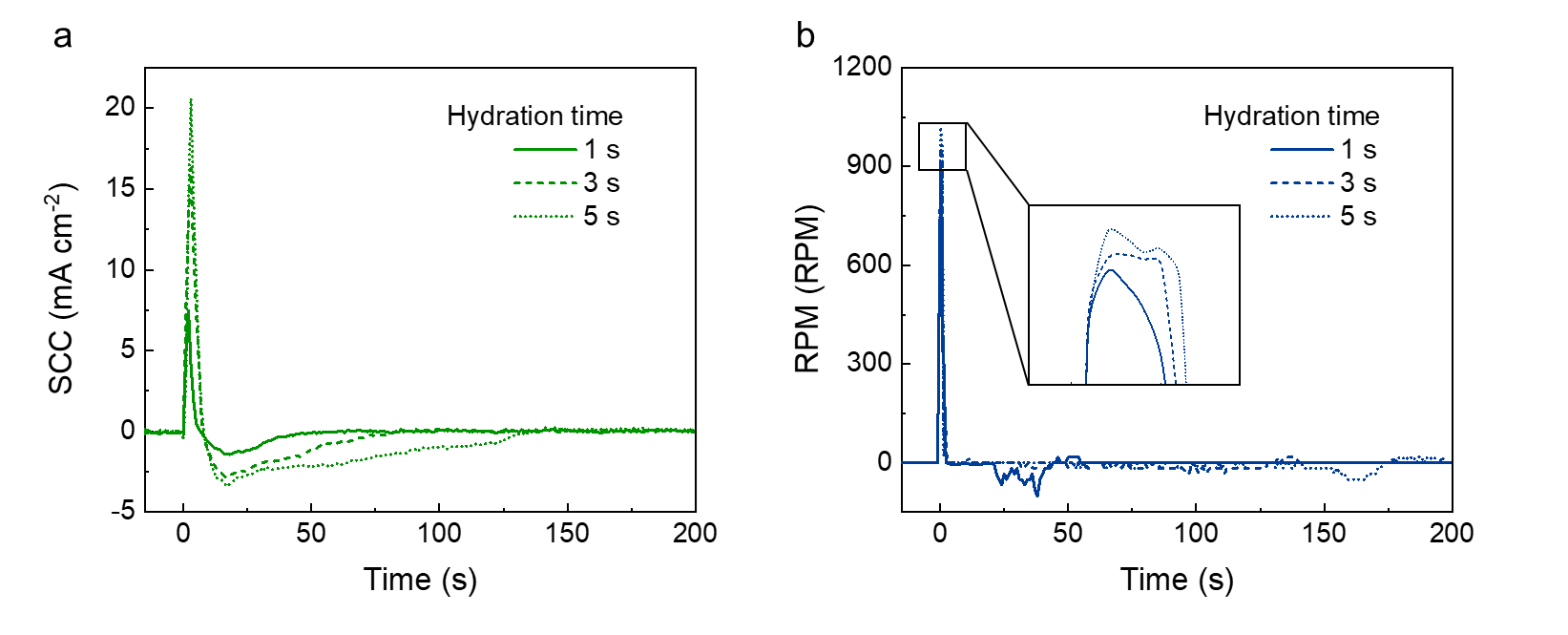


**Figure S15** Time dependences of a) short-circuit current (SCC) and b) rotation per minute (RPM) for a heterophilic CNT yarn under various hydration times 1, 3, and 5 s.

**
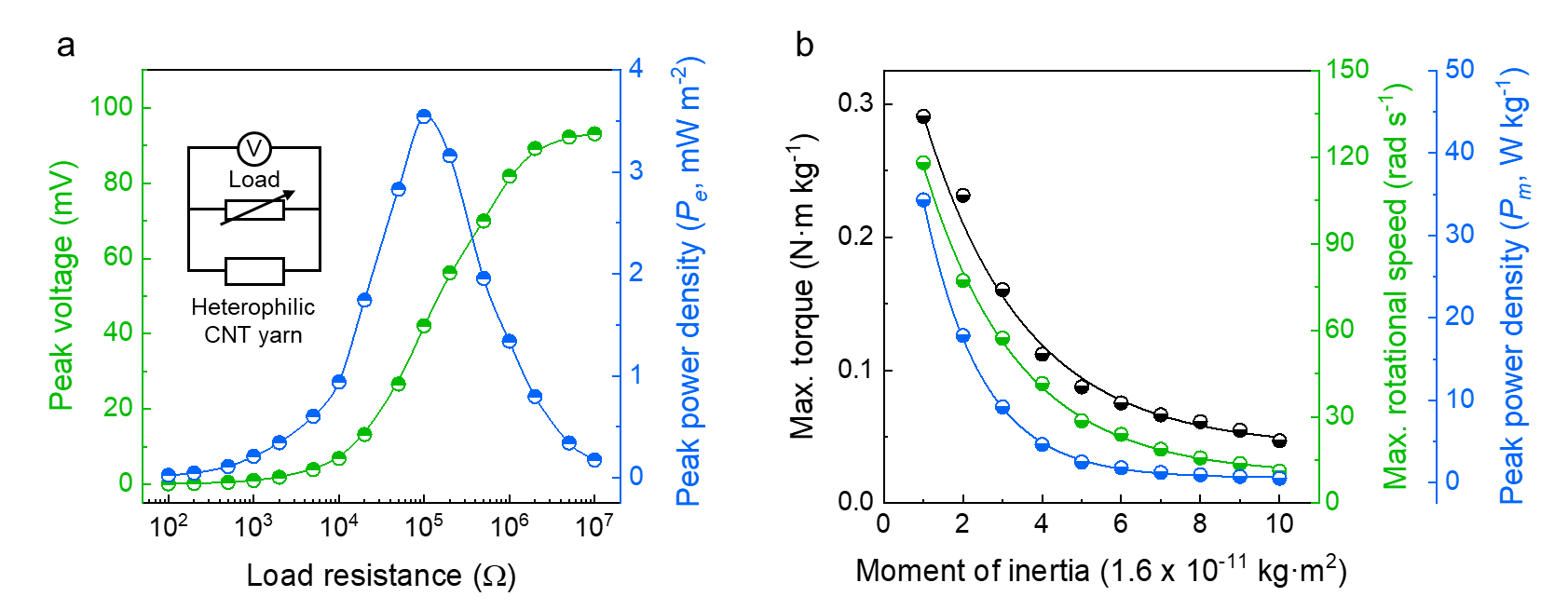
**

**Figure S16** a) Generated peak voltage and electrical peak power density as a function of external load resistance. Inset: An schematic illustration of the experimental circuit model used for electrical power estimation. (b) Maximum torque, rotational speed, and mechanical peak power density as a function of moment of inertia of attached paddle.

**
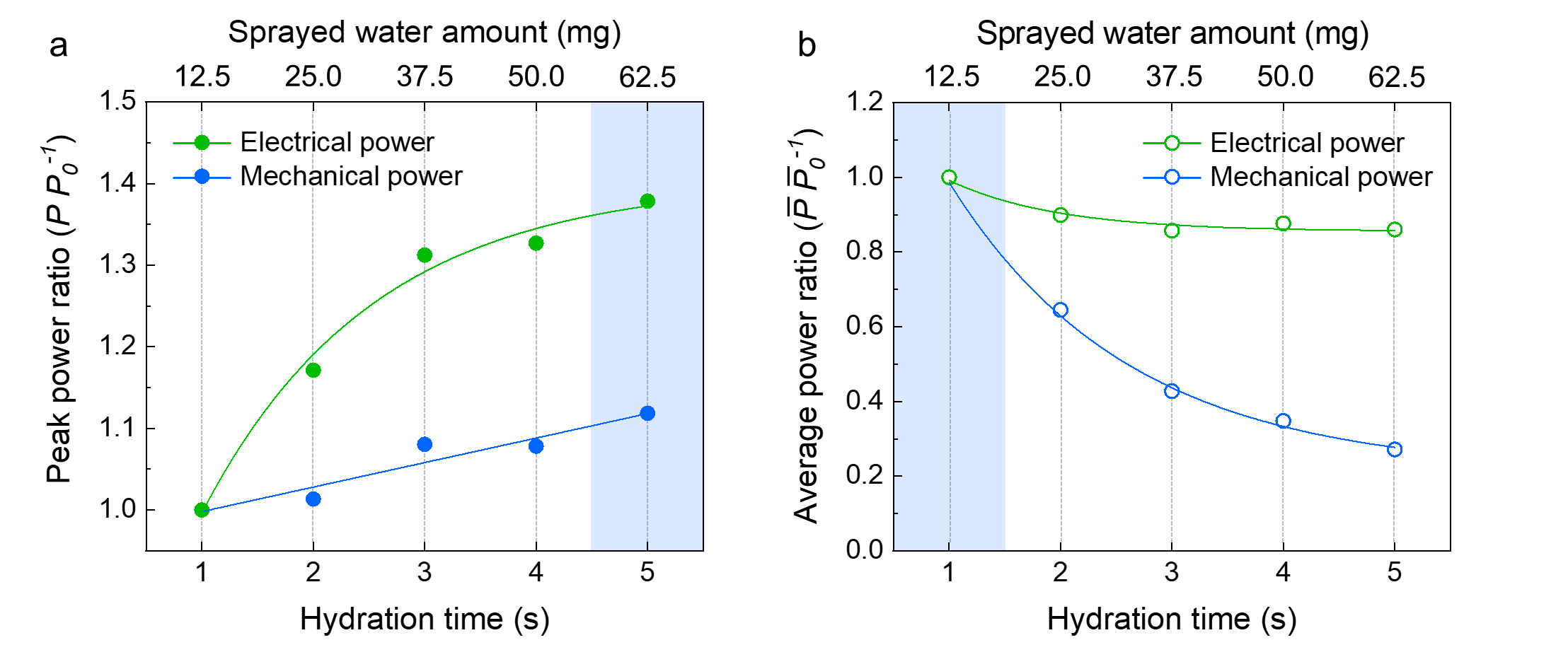
**

**Figure S17** a) Peak power density (*P*) and b) average power density (*P̄*) ratio of electrical and mechanical torsional harvesting as a function of hydration time from 1 to 5 s. The blue shaded regions indicate the optimal sprayed water conditions.

**
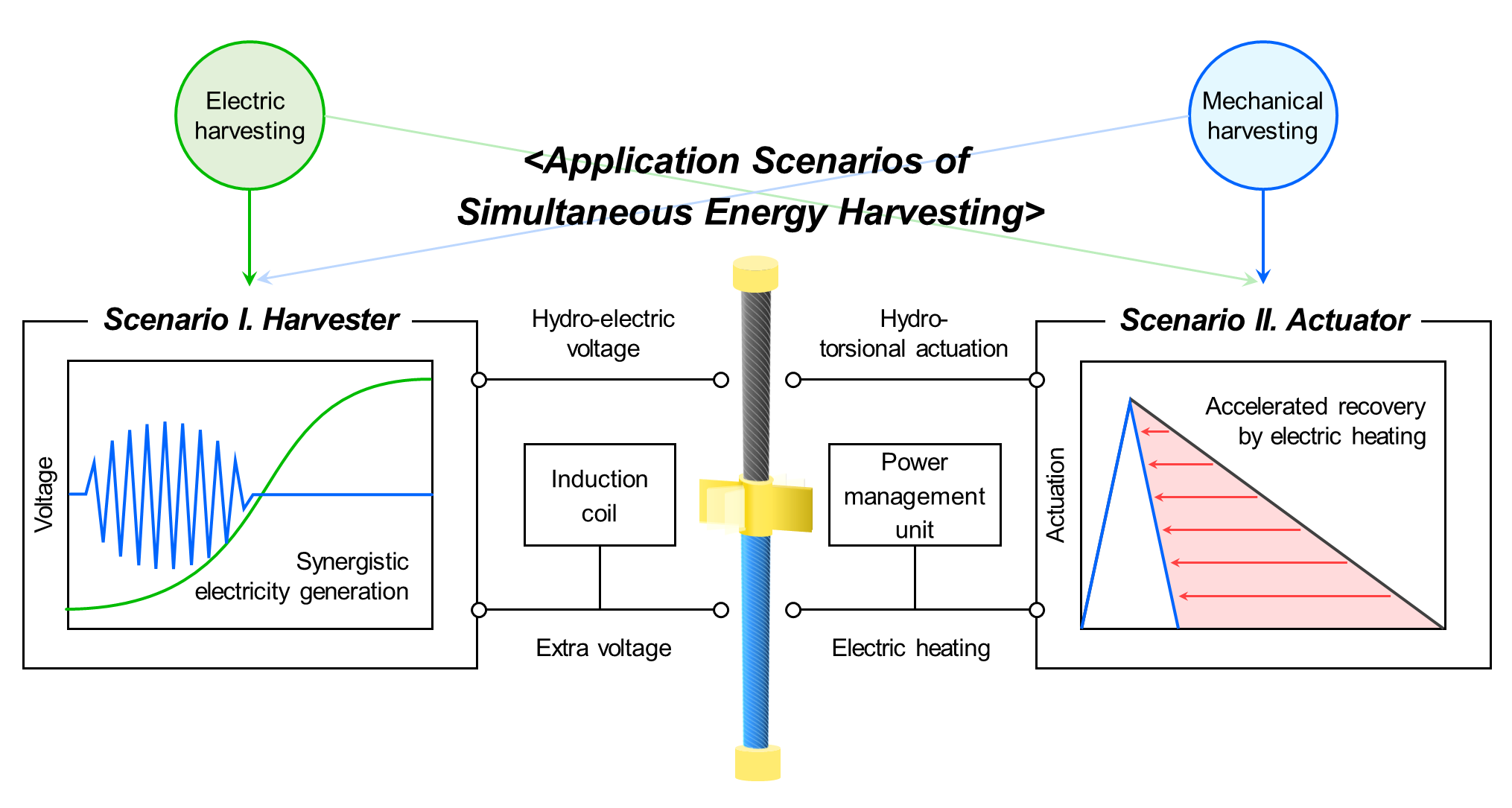
**

**Figure S18.** Schematic illustration depicting the sustainable application scenarios of simultaneous energy haversting generated from heterophilic CNT yarn, with each focusing on an electrical energy harvester (Scenario I) and a mechanical actuator (Scenario II).


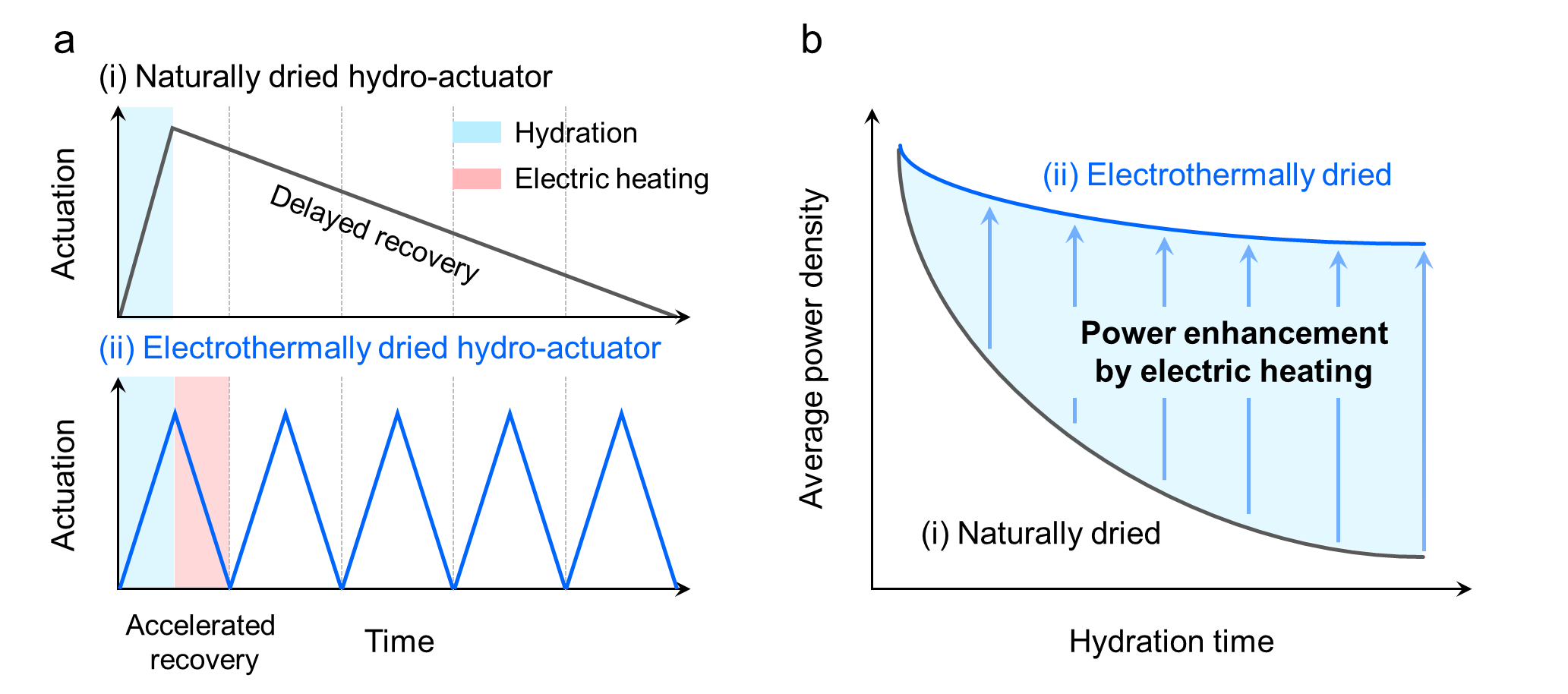


**Figure S19** a) Expected actuation profiles of naturally dried (upper panel) and electrothermally dried (lower panel) hydro-actuators. b) Expected power enhancement effect enabled by electric heating.

**Table S1.** Binding energy and content of each functional group calculated from the deconvoluted C1s spectra.

|  | **C=C** | | **C-C** | | **C-OH** | | **C-O-C** | | **COOH** | | **π-π** | | **O/C**  **Ratio** |
| --- | --- | --- | --- | --- | --- | --- | --- | --- | --- | --- | --- | --- | --- |
|  | Peak  (eV) | Content  (%) | Peak  (eV) | Content  (%) | Peak  (eV) | Content  (%) | Peak  (eV) | Content  (%) | Peak  (eV) | Content  (%) | Peak  (eV) | Content  (%) |  |
| **HPB** | 284.3 | 62.5 | 285.0 | 21.6 | 285.7 | 6.6 | 286.8 | 4.1 | 288.3 | 2.0 | 289.7 | 3.2 | 0.03 |
| **HPL** | 284.4 | 39.4 | 284.8 | 21.7 | 286.4 | 18.7 | 287.7 | 11.7 | 288.8 | 4.5 | 289.1 | 4.0 | 0.20 |

**Table S2** Comparison of yarn configuration and electrical and mechanical harvesting performances of heterophilic CNT yarn with previous yarn- or fiber-structured hydro-electric harvesters and torsional hydro-actuator.

| **Harvesting**  **mode** | **Yarn configuration**  **[Ref. No.]** | **Hydro-electric harvester** | | **Hydro-torsional actuator** | | |
| --- | --- | --- | --- | --- | --- | --- |
|  |  | **OCV**  **[mV]** | **Electrical power***  **[mW m^-2^]** | **Voltage**†  **[mV]** | **Stroke**  **[^o^ mm^-1^]** | **Mechanical power**  **[W kg^-1^]** |
| **Dual-mode** | **Heterophilic CNT yarn**  **[This work]** | **106** | **3.5** | **13.6** | **78.8** | **34.3** |
| Single-mode | GO/Ag co-axial wire^[1]^ | 300 | - | *(not addressed)* | | |
|  | CNT/Ecoflex buckled yarn^[2]^ | 80 | 2.5 |  |  |  |
|  | GO/Hydrogel yarn^[3]^ | *(not addressed)* | | 1 | 588 | 71.9 |
|  | Alginate coiled yarn^[4]^ |  |  | 18 | 756 | - |
|  | Viscose/PET plied yarn^[5]^ |  |  | 5 | 581 | - |

*Electrical power density was calculated at the optimal external resistance for maximum output

†Electromagnetically induced voltage

**References**

[1] C. Shao, J. Gao, T. Xu, B. Ji, Y. Xiao, C. Gao, Y. Zhao, L. Qu, *Nano Energy*, **2018**, *53*, 698–705.

[2] H. J. Sim, H. Gwa, S. J. Kim, J.-M. Oh, C. Choi, *Chem. Eng. J.* **2024**, *495*, 153486.

[3] H. Cheng, Y. Hu, F. Zhao, Z. Dong, Y. Wang, N. Chen, Z. Zhang, L. Qu, *Adv. Mater.* **2014**, *26*, 2909–2913.

[4] W. Wang, C. Xiang, Q. Liu, M. Li, W. Zhong, K. Yan, D. Wang, *J. Mater. Chem. A* **2018**, *6*, 22599–22608.

[5] N. Sheng, Y. Peng, F. Sun, J. Hu, *Adv. Fiber. Mater.* 2023, **5**, 1534.
